# Modeling Phototaxis and Planktonic Cell Behavior in Phototrophic Biofilms

**DOI:** 10.1007/s11538-026-01753-w

**Published:** 2026-09-23

**Authors:** Alberto Tenore, Fabiana Russo, Luigi Frunzo, Maria Rosaria Mattei

**Affiliations:** Department of Mathematics and Applications “Renato Caccioppoli”, University of Naples Federico II, Via Cintia, Monte S. Angelo, 80126 Naples, Italy

**Keywords:** Phototaxis, Phototrophic biofilms, 2-D Modeling, Numerical simulations

## Abstract

We propose a continuous multidimensional multispecies model of planktonic cell invasion in phototrophic biofilms that incorporates phototaxis as a key mechanism driving directed movement along light gradients. The biofilm is modeled as a homogeneous, viscous, incompressible fluid, with the velocity field governed by a Darcy-type law. The phototactic behavior of planktonic cells is integrated in the model by considering a reaction–advection–diffusion equation including a diffusive and tactic flux with a volume-filling term. Cell movement toward favorable light conditions and away from harmful ones is modeled through a light-dependent sensitivity function capturing both positive and negative phototaxis. Planktonic cells are attracted to regions with optimal light intensity, where they accumulate as an effect of phototactic behavior, and might switch their phenotype from planktonic to sessile, contributing to biofilm expansion. Numerical simulations highlight the crucial role of phototaxis in biofilm dynamics, showing how the balance between random diffusion and phototactic movement regulates biomass distribution and, consequently, oxygen and organic carbon dynamics. Under high-light stress conditions, photoinhibition reverses phototaxis, causing planktonic cells to move away from the light source, reducing overall biofilm development. Higher biofilm densities increase light attenuation, which, depending on light conditions, either reduces overall phototrophic growth or provides protection against excessive light exposure. Finally, biofilm geometry influences light attenuation and phototrophic growth, with its effects depending on incident light conditions and biofilm density. Overall, the proposed multispecies framework provides a mechanistic tool to investigate how phototactic motility, light conditions, biofilm properties, and microbial interactions jointly regulate phototrophic biofilm development.

## Introduction

Motility is a vital feature in the life cycle of most bacteria, enabling them to access essential resources and colonize environments ideal for their growth and proliferation (Wadhwa and Berg 2022). Although bacterial movement might appear random at first glance, research conducted over a century ago (Engelmann 1882; Pfeffer 1888) uncovered that certain bacteria are capable of directed movement in response to external stimuli, a phenomenon recognized as bacterial taxis. This behavior enables bacteria to navigate their environment by either moving toward favorable stimuli, called attractants, or away from harmful ones, known as repellents. Based on the direction of movement, bacterial taxis is classified into positive taxis, where bacteria move toward attractants, and negative taxis, where they move away from repellents (Adler 1975). This ability allows bacteria to adapt to and thrive in dynamic and diverse environments.

Taxis has been observed in response to a variety of environmental stimuli, including metabolites, signaling molecules, temperature, light, salinity, oxygen, magnetic fields, and pH (Krell et al. 2011). Among these, chemotaxis, the movement toward or away from chemical gradients, is one of the most studied and critical forms of taxis, as it allows organisms to locate essential nutrients or avoid harmful substances (Wadhwa and Berg 2022). Likewise, phototaxis refers to the directed movement of microorganisms in response to light, where they either approach or move away from a light source. This behavior is commonly observed in microorganisms that are predominantly photosynthetic (Clayton 1964), allowing them to adjust their position to enhance light capture for photosynthetic processes (Bhaya 2004).

Bacterial cells typically exhibit two different phenotypes: the planktonic state which is characterized by a significantly higher motility compared to the sessile counterpart, where bacteria live attached to abiotic or biotic surfaces, surrounded by a self-produced matrix, known as a biofilm (Guillín et al. 2025). Sessile cells suppress motility-related gene expression in favor of matrix production, prioritizing structural stability and cooperative interactions within the community (Colin et al. 2021). Biofilm communities are significantly shaped by the interplay of the two phenotypes, in an alternating cycle in which planktonic cells transition to sessile cells within a biofilm and sessile cells detach from the biofilm to planktonic cells, as a result of differential gene expression and cellular communication mechanisms. Despite the reduced motility of its individual sessile inhabitants, biofilms serve as a crucial habitat that encourages the taxis-based movement of planktonic cells (Muhammad et al. 2020). While sessile cells remain largely stationary within the biofilm matrix, planktonic cells are able to adjust their position, actively navigating through the pores and channels of the biofilm’s dense extracellular polymeric matrix (Oliveira et al. 2016). This motility is not only a distinguishing feature but also a critical factor influencing biofilm ecosystem dynamics. It enables bacteria to access nutrient-rich microenvironments or oxygenated zones within the biofilm, optimizing their metabolic activity. As a result, the dynamic interplay between planktonic and sessile cells can substantially reshape the spatial organization of the biofilm, alter nutrient gradients, and impact its overall functionality, emphasizing the vital ecological role of bacterial motility (Brileya et al. 2014). Furthermore, this insight suggests the potential for systematically engineering biofilms by manipulating the movement of planktonic cells (Oliveira et al. 2016). A relevant case can be represented by phototrophic biofilms where light is essential for driving photosynthetic processes. In particular, the behavior of planktonic cells in phototrophic biofilms can be strongly affected by phototaxis, which becomes relevant in the context of photobioreactors or natural systems, where light distribution is often non-uniform (Ogbonna and Tanaka 2000; Strieth et al. 2018).

Mathematical modeling of bacterial taxis has long stood out as a central topic in mathematical biology, inspiring an extensive body of theoretical research aimed at understanding this phenomenon. Reviews by Horstmann (2003) and Hillen and Painter (2009) highlight the diversity of approaches developed to describe chemotaxis, including models based on Fourier’s and Fick’s laws, biased random walks, interacting particle systems, transport equations, stochastic processes, and multi-phase flow frameworks. Among these, the Keller-Segel model (Keller and Segel 1970, 1971) is widely recognized as a cornerstone in chemotaxis research. This deterministic model employs reaction–advection–diffusion equations to describe cell movement as a combination of diffusion and directed motion toward regions of higher attractant concentration. The Keller-Segel model is particularly well-suited for capturing large-scale population dynamics and explaining key phenomena such as aggregation and pattern formation in bacterial populations, establishing it as a foundational tool in the field. Importantly, Hillen and Painter (2001), and Painter et al. (2002) introduced a “volume filling” approach, incorporating a biologically realistic population-sensing mechanism into the Keller–Segel model to prevent finite time blow-up and “overcrowding” scenarios.

Regarding phototactic motility, several mathematical models have been proposed. Early works include the combined cellular automata–PDE model by Marée et al. (1999) and the framework of Burkart and Häder (1980), which explained phototactic attraction in microorganisms but neglected cell–cell interactions. Later, agent-based approaches incorporated interactions mediated by light transmission between cells (Fatehi et al. 2010). Experimental observations by Burriesci and Bhaya (2008) on Synechocystis sp. PCC 6803 showed that collective behavior enhances motility and produces finger-like projections, motivating stochastic particle models by Bhaya et al. (2008), Levy and Requeijo (2008a, 2008b) and hierarchical descriptions by Ha and Levy (2009). More recently, active-matter models have been introduced to reproduce cell trajectories and colony morphologies, accounting for probabilistic light sensing, slime deposition, and dynamic neighbor interactions (Varuni et al. 2017; Menon et al. 2020, 2021). Finally, Giometto et al. (2015) showed that the Keller–Segel framework can capture both positive and negative phototaxis when phototactic sensitivity follows a generalized receptor law describing nonlinear responses to light intensity.

None of these models, though, address phototaxis in biofilm ecosystems. Building on this gap, the aim of our work is to present a model that captures how phototaxis of planktonic cells influences phototrophic biofilm dynamics, ecology, and microbial species competition. The model we propose employs a continuous framework, incorporating the dynamics of planktonic cell invasion into Alpkvist and Klapper’s multidimensional biofilm model (Alpkvist and Klapper 2007). Specifically, the phototactic behavior of planktonic cells is accounted for by incorporating a tactic flux with a volume-filling term into the invasion equation originally proposed by D’Acunto et al. (2015), thereby extending the model to capture directed motility in addition to the previously assumed purely random movement. The phototactic sensitivity function, which depends on light intensity, effectively captures both positive and negative phototaxis within a unified mathematical framework, describing cell movement toward favorable light conditions and away from harmful ones. Additionally, the model accounts for the presence of heterotrophic species and their interactions with phototrophs, including competition for space, nutrient exchange, and metabolic cooperation - processes that are widely studied in biofilm modeling (Wolf et al. 2007; Muñoz Sierra et al. 2014; Tenore et al. 2021, 2024) - which further influence biofilm structure and function.

The work is organized as follows. Section 2 presents the mathematical framework describing bacterial phototaxis in phototrophic biofilms. Different numerical studies were conducted in Sect.  3, investigating the effect of: random and phototactic motility conditions (Sect.  3.1); light conditions (Sect.  3.2); biofilm density (Sect.  3.3); initial biofilm geometry (Sect.  3.4). The comparison in Sect.  3.1 allows for the assessment of how phototactic behavior influences the spatial distribution and overall dynamics of the microbial population. In Sect.  3.2, two light conditions were tested: an optimal light intensity for phototactic response and an excessively high incident light intensity, to investigate the model’s behavior under both positive and negative phototaxis conditions. Furthermore, the numerical study in Sect.  3.3 was conducted to evaluate the impact of biofilm density on the system’s dynamics. By varying the biofilm density, the model provides insights into how cell aggregation influences light penetration, phototactic responses, and the spatial distribution of planktonic cells. The last numerical study presented in Sect.  3.4 assesses the influence of the initial biofilm geometry on model predictions. Section  3.5 summarizes the effects of the investigated parameters on planktonic cell mass. Finally, Sect.  4 discusses the main findings and implications of the study. The results show that phototaxis plays a central role in shaping the spatial distribution of planktonic cells and promoting phototrophic biofilm development, with the balance between random and phototactic motility strongly influencing microbial spatial organization. The analysis of light conditions shows that optimal illumination promotes phototrophic growth and positive phototaxis, whereas excessive light can induce photoinhibition, leading to negative phototaxis and slower biofilm development. Furthermore, biofilm density and geometry modulate light attenuation and, consequently, the local light conditions experienced by microbial cells, thereby influencing phototactic migration and biofilm growth. Overall, these findings highlight the importance of phototactic behavior in biofilm establishment and suggest its potential relevance to the start-up of biofilm-based photobioreactors. The section also outlines the main limitations of the model and possible extensions.

## Model Framework

This section presents a mathematical model describing bacterial taxis of motile cells within multispecies biofilm communities. Building on the multidimensional biofilm model proposed by Alpkvist and Klapper (2007), we extend it by integrating the dynamics of motile cells, including their tactic behavior. This approach enables a detailed characterization of motile cell movement within these microbial communities and their influence on the biofilm structure and dynamics.

As a specific application, we investigate the role of phototaxis in phototrophic biofilms. Phototaxis plays a crucial role in the behavior and development of biofilms composed of light-sensitive microorganisms. By incorporating phototactic behavior of planktonic cells into the established biofilm framework, we can deeper investigate how microorganisms in phototrophic biofilms move relative to light sources, ultimately influencing biofilm structure, growth patterns, and nutrient uptake. This understanding is important in various contexts, including the optimization of photobioreactors, where efficient light utilization is essential for maximizing microbial productivity (Ogbonna and Tanaka 2000), as well as in environmental and industrial applications where biofilm formation and light-driven processes are crucial (Strieth et al. 2018). In this perspective, the system considered hereafter can be interpreted as a photobioreactor. Accordingly, the model setup was selected to reflect this setting and will be treated consistently in the following.

**Fig. 1:**
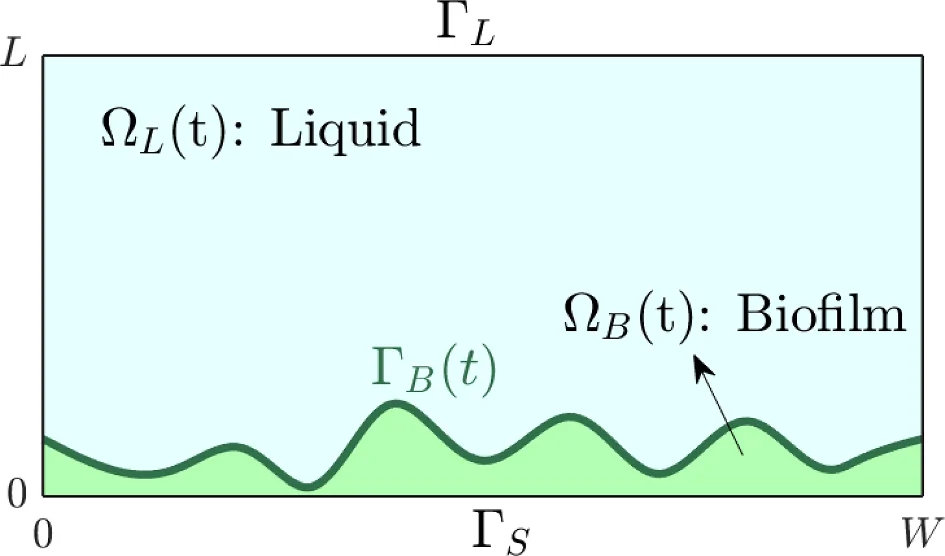
Two-dimensional illustration of the domain Ω with subsets $\Omega _B(t)$ (biofilm, in green) and $\Omega _L(t) = \Omega \backslash \Omega _B(t)$ (liquid, in light blue), separated by the biofilm-liquid interface $\Gamma _B(t)$. The top and bottom boundaries are denoted by $\Gamma _L$ and $\Gamma _S$, respectively. *W* = 2 mm and *L* = 1 mm (Color figure online)

**Fig. 2:**
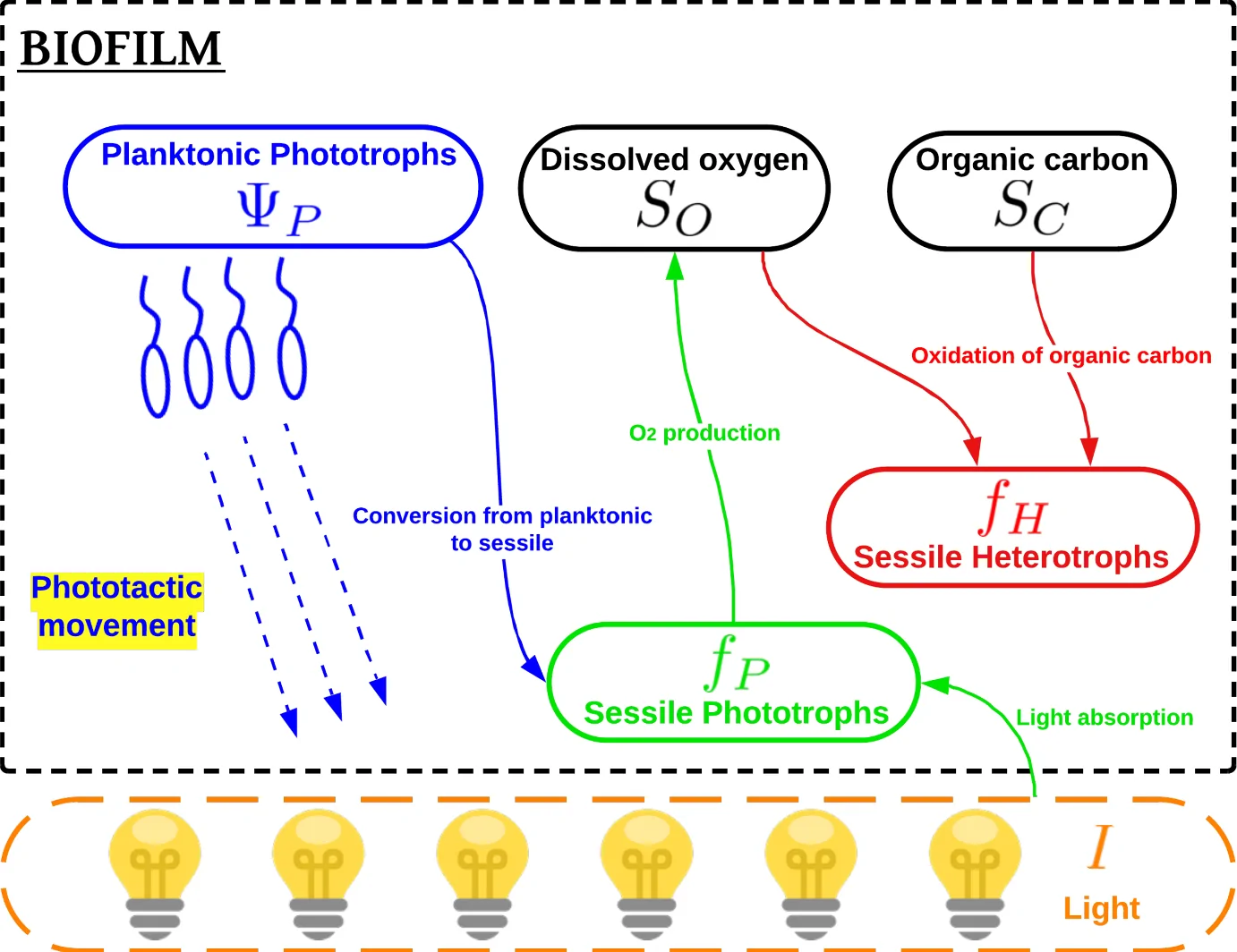
Schematic representation of the model biological pathways. Created in Lucidchart (www.lucidchart.com) (Color figure online)

As shown in Fig.  1, we consider a two-dimensional domain:

1 $$\begin{aligned} \Omega = \{{\textbf {x}}=(x,y), \ 0< x< W, \ 0< y < L \}, \end{aligned}$$

where *W* and *L* denote the fixed width and length of the domain, respectively. This domain consists of two distinct regions: the biofilm region $\Omega _B(t)$ and the liquid region $\Omega _L(t) = \Omega \backslash \Omega _B(t)$. These regions are separated by the curve $\Gamma _B(t)$, which represents the biofilm-liquid interface defined by y=h(x,t). The top boundary $\Gamma _L$ is in contact with a bulk liquid with prescribed properties. The bottom boundary $\Gamma _S$ is represented by a flat, impermeable substratum, which also serves as the light source.

The biofilm region $\Omega _B(t)$ is populated by two sessile microbial species, phototrophs *P* and heterotrophs *H*, whose concentrations are denoted by $X_i(\textbf{x},t)$, with $i \in \{P,H\}$, and planktonic phototrophs, whose concentration is denoted by $\Psi _P(\textbf{x},t)$. Planktonic phototrophs exhibit phototactic behavior and can transition from the planktonic to the sessile phenotype. The effects of light on both phototrophic metabolic activity and cell motility are represented through the light intensity $I(\textbf{x},t)$. Finally, the model includes two dissolved substrates, oxygen *O* and organic carbon *C*, whose concentrations are denoted by $S_j(\textbf{x},t)$, with $j \in \{O,C\}$. These substrates diffuse throughout the entire biofilm-liquid domain Ω and play a key role in sustaining microbial activity and governing the overall dynamics of the phototrophic biofilm. We further assume that:

- Sessile components have the same density and are incompressible, implying that their density is constant at position ${{\textbf {x}}}$ and time *t* (Wanner and Gujer 1986), i.e., $\rho ({{\textbf {x}}},t) = \rho , \ {{\textbf {x}}} \in \Omega _B(t), \ t \in [0,T]$.
- The volume occupied by planktonic cells within the biofilm is neglected, as their overall volume fraction is assumed to be negligible compared with that of the biofilm matrix. They contribute to biofilm growth (i.e., biofilm volume increase) only after phenotype switching, when they become part of the solid phase (D’Acunto et al. 2015, 2018, 2019, 2021). This approximation is justified because motile phototactic (planktonic) cells are typically only a few micrometers in size (Schuergers et al. 2017; Montgomery 2015; Bozan et al. 2025), whereas the biofilm’s cells and matrix (sessile components) occupy much larger spatial scales.
- Since the sessile cells are uniformly distributed with fixed density, proliferation gives rise to an internal pressure which results in the movement of sessile cells.

The volume fractions of the sessile species can be determined by dividing the biomass concentration $X_i({{\textbf {x}}},t)$ by the density ρ (Wanner and Gujer 1986), yielding $f_i({{\textbf {x}}},t) = X_i({{\textbf {x}}},t) / \rho$, where $i \in \{ P, H \}$, and ${{\textbf {x}}} \in \Omega _B(t)$ and t∈[0,T] represent the spatial and temporal domains of the biofilm. All volume fractions $f_i$ are constrained to sum to one at each point in space and time (Wanner and Gujer 1986): $f_P({{\textbf {x}}},t)+f_H({{\textbf {x}}},t) = 1$ for ${{\textbf {x}}} \in \Omega _B(t)$ and t∈[0,T].

An overview of the model variables and processes is illustrated in Fig. 2. The light emitter, providing a constant light intensity $I_0$ is assumed to be located at y=0, and the light intensity $I({\textbf {x}},t)$ attenuates vertically through the biofilm according to Lambert-Beer law (Tenore et al. 2021):

2 $$\begin{aligned} I=I_0 e^{-\rho k_{tot}y}, \end{aligned}$$

where $k_{tot}$ is the biofilm attenuation coefficient. This exponential attenuation accounts for the absorption and scattering of light by the biofilm constituents, influencing both the growth rates and motility patterns of phototrophic organisms. Sessile phototrophs $f_{P}$ use light *I* as an energy source for growth and release oxygen $S_{O}$, which is consumed by heterotrophs $f_{H}$ to oxidize organic carbon $S_{C}$ during their metabolism. The phototactic movement of planktonic phototrophs $\Psi _P$ and their transition to the sessile phenotype $f_P$ are regulated by the light intensity *I*.

Based on the biological framework outlined above, the mathematical model is formulated as a system of governing equations derived from mass conservation principles and supplemented by suitable initial and boundary conditions. For the sessile species, this leads to the following equations:

3 $$\begin{aligned} \partial _t f_P + \underbrace{\nabla \cdot \big ({\textbf {u}} f_P\big )}_{ \begin{array}{c} \text {advective}\\ \text {transport} \end{array} }&= \underbrace{r_{M,P}\big (f_P, I\big )}_{\text {growth}} + \underbrace{r_{P}\big (\Psi _P, I\big )}_{ \begin{array}{c} \text {switching from}\\ \text {planktonic to sessile} \end{array} }, \ {\textbf {x}} \in \Omega _B(t), \ t \in ]0,T], \end{aligned}$$

4 $$\begin{aligned} \partial _t f_H + \underbrace{\nabla \cdot \big ({\textbf {u}} f_H\big )}_{ \begin{array}{c} \text {advective}\\ \text {transport} \end{array} }&= \underbrace{r_{M,H}\big (f_H, S_O, S_C\big )}_{\text {growth}}, \ {\textbf {x}} \in \Omega _B(t), \ t \in ]0,T], \end{aligned}$$

where $r_{M,P}$ and $r_{M,H}$ are the growth rates associated with the proliferation of pre-existing sessile species *P* and *H*, respectively; and $r_{P}$ is the growth rate due to planktonic-to-sessile switching of phototrophs. Following the continuum multispecies biofilm framework introduced by Alpkvist and Klapper (2007), a Darcy-type relation is adopted for the common growth-induced velocity of the biofilm continuum, ${\textbf {u}}={\textbf {u}}({\textbf {x}},t)$, shared by all sessile biomass components:

5 $$\begin{aligned} {\textbf {u}} = -\lambda _p \nabla p, \end{aligned}$$

where $p=p({\textbf {x}},t)$ is the pressure and $\lambda _p>0$ is the Darcy constant. By summing Eq. (3) and Eq. (4), and using Eq. (5) together with the volume-fraction constraint $f_P+f_H=1$, we derive the following equation for $p({\textbf {x}},t)$:

6 $$\begin{aligned} \nabla \cdot {\textbf {u}} = - \lambda _p \nabla ^2 p = r_{M,P} + r_{P} + r_{M,H}, \ {\textbf {x}}\in \Omega _B(t), \ t \in [0,T]. \end{aligned}$$

The divergence of the biofilm velocity is therefore entirely determined by the local biomass production rates, consistently with the saturation constraint of the biofilm continuum.

The biofilm-liquid interface y=h(x,t) moves according to:

7 $$\begin{aligned} \frac{dh}{dt} = - \lambda _p (\nabla p \cdot {\textbf {n}})(\hat{\textbf{y}} \cdot {\textbf {n}}), \end{aligned}$$

where $\hat{\textbf{y}}$ is a unit vector in the *y*-direction.

As observed in Eq. (6), *p* is proportional to $\lambda _p^{-1}$. Hence, the biofilm velocity ${\textbf {u}}$ is independent of $\lambda _p$ (Klapper 2004; Alpkvist and Klapper 2007). As a result, no specific value needs to be prescribed for $\lambda _p$, since different choices simply correspond to equivalent rescalings of the pressure field while leaving the velocity field and the predicted biofilm evolution unchanged.

The reaction terms associated with Eqs. (3) and (4) are defined as:

8 $$\begin{aligned} r_{M,P} = \mu _{max,P} \ \frac{I}{I_{\text {opt}}} \ e^{1-\frac{I}{I_{\text {opt}}}} \ f_{P}, \end{aligned}$$

9 $$\begin{aligned} r_{P} = k_{col} \ \frac{I}{I_{\text {opt}}} \ e^{1-\frac{I}{I_{\text {opt}}}} \ \frac{\Psi _{P}}{\rho }, \end{aligned}$$

10 $$\begin{aligned} r_{M,H} = \mu _{max,H} \ \frac{S_{C}}{K_{C}+S_{C}} \ \frac{S_{O}}{K_{O}+S_{O}} \ f_{H}, \end{aligned}$$

where $\mu _{max,P}$ and $\mu _{max,H}$ are the maximum specific growth rate of phototrophic and heterotrophic sessile species; $k_{col}$ is the conversion rate from planktonic to sessile; $I_{\text {opt}}$ is the optimal light intensity for phototrophic growth; $K_{C}$ and $K_{O}$ are the half-saturation constants for carbon and oxygen. Notably, the phototrophic growth rate is not a monotonic function of light intensity. Under excessively high light intensities, the photosynthetic machinery of cells can be partially damaged, a phenomenon known as photoinhibition, which reduces the efficiency of their metabolic processes (Tilzer 1987). Accordingly, the formulation adopted for the light-dependent growth rate incorporates the effects of photoinhibition, thereby limiting phototrophic growth under excessively high light conditions (Liehr et al. 1990). In contrast, $r_{M,H}$ is modeled using saturation Monod-type kinetics, with oxygen and organic carbon serving as substrates for heterotrophic growth (Henze et al. 2006).

The dynamics of planktonic phototrophs are described by the following equation:

11 $$\begin{aligned} \begin{aligned} \partial _t \Psi _P + \nabla \cdot&\big (\underbrace{-D_{\Psi } \nabla \Psi _P}_{\text {random movement}} \! + \! \underbrace{{\Psi _P \textbf{v}}}_{\text {tactic movement}}\big ) \! = \! \underbrace{- \rho \ \! r_{P}\big (\Psi _P, I\big )}_{\text {planktonic-to-sessile switch}},\\&\ {\textbf {x}} \in \Omega _B(t), \ t \in ]0,T], \end{aligned} \end{aligned}$$

where ${\textbf {v}}={\textbf {v}}({\textbf {x}},t)$ represents the tactic velocity of planktonic phototrophs, while $D_{\Psi }$ is the diffusion coefficient in the biofilm region. This formulation captures both advective and diffusive processes. The diffusive flux accounts for the random movement of cells, as introduced in D’Acunto et al. (2015), while the advective flux describes bacterial taxis, representing directed movement in response to environmental cues. The advective transport of planktonic species is modeled using a volume-filling approach, which accounts for density-dependent sensitivity (Hillen and Painter 2001; Painter et al. 2002; Hillen and Painter 2009):

12 $$\begin{aligned} \mathbf{{v}}({\textbf {x}},t)=\frac{\chi _{1}}{\chi _{2} + \Psi _P({\textbf {x}},t)} \nabla \Phi ({\textbf {x}},t), \end{aligned}$$

where $\chi _{1}$ and $\chi _{2}$ are positive constants related to the sensitivity and saturation of the species’ response to the stimulus, respectively. The incorporation of a “volume-filling” term aids in preventing finite time blow-up and “overcrowding” situations. $\nabla \Phi$ is the gradient of the phototactic function Φ. Photoinhibition also influences the phototactic behavior of microorganisms, inducing negative phototaxis—where cells actively move away from high-light stress zones toward regions with reduced light intensities (Raven 2011). To model this phenomenon, we chose a phototactic function Φ that captures both positive and negative tactic responses:

13 $$\begin{aligned} \Phi = \frac{I}{I_{\text {opt}}} \ e^{1-\frac{I}{I_{\text {opt}}}}. \end{aligned}$$

Notably, the effect of light intensity on both the phototactic movement of motile cells (in Eq. (13)) and the metabolic activity of phototrophs (in Eqs. (8) and (9)) is modeled using the same exponential function. This reflects the idea that motile cells migrate toward regions where they can grow faster.

Transport of dissolved substrates within both the biofilm and liquid is governed by Fick’s laws:

14 $$\begin{aligned} \partial _t S_j -&\underbrace{D^B_{S,j} \nabla ^2 S_j}_{\text {diffusive transport}} = \underbrace{r_{S,j}({\textbf{f}},\textbf{S},I)}_{\text {conversion}}, \ j \in \{ O, C \}, \ {\textbf {x}} \in \Omega _B(t), \ t \in ]0,T], \end{aligned}$$

15 $$\begin{aligned} \partial _t S_j -&\underbrace{D^L_{S,j} \nabla ^2 S_j}_{\text {diffusive transport}} = 0, \ j \in \{ O, C \}, \ {\textbf {x}} \in \Omega \backslash \Omega _B(t), \ t \in ]0,T], \end{aligned}$$

where $\mathbf{{f}}=(f_P,f_H)$, $\mathbf{{S}}=(S_O, S_C)$; $D^B_{S,j}$ and $D^L_{S,j}$ denote the diffusivities of substrate *j* within the biofilm and the liquid phase respectively; $r_{S,j}$ is the conversion rate for nutrients in the biofilm region. The transport dynamics of dissolved substrates in the liquid region is described by a homogeneous system of equations, reflecting the assumption that biological conversion processes are negligible within this region. The conversion rates of substrates $r_{S,j}$, with $j \in \{ O, C \}$ are defined as:

16 $$\begin{aligned} r_{S,C} = - \frac{1}{Y_H} \ r_{M,H} \ \rho , \end{aligned}$$

17 $$\begin{aligned} r_{S,O_2} = \frac{1}{PM_O} \ r_{M,P} \ \rho - \frac{1}{PM_O} \bigg (\frac{1}{Y_H}- 1 \bigg ) \ r_{M,H} \ \rho , \end{aligned}$$

where $Y_H$ is the yield coefficient of heterotrophic growth and $PM_O$ denotes the molecular weight of oxygen, introduced to convert oxygen from moles to grams of COD, ensuring consistency of units. As depicted in Fig.  2, organic carbon $S_C$ is consumed by heterotrophs, whereas oxygen $S_O$ is produced by phototrophs and consumed by heterotrophs.

**Table 1:** Model parameters and boundary values

Parameter	Definition	Unit	Value	Ref
ρ	Biofilm density	g(COD) $\text {m}^{-3}$	$37000^{ \ (a)}$	Muñoz Sierra et al. (2014)
$\mu _{max,P}$	Maximum growth rate of $f_{P}$	$\hbox {d}^{-1}$	$2^{\ (b)}$	Reichert et al. (2001)
$\mu _{max,H}$	Maximum growth rate of $f_{H}$	$\hbox {d}^{-1}$	$4.8^{\ (b)}$	Wanner and Gujer (1986)
$K_{C}$	*C* half saturation coefficient	g(COD) $\hbox {m}^{-3}$	4	Boltz et al. (2011)
$K_{O}$	$\hbox {O}_2$ half saturation coefficient	mol($\hbox {O}_2$) $\hbox {m}^{-3}$	3.125$\cdot 10^{-3}$	Wanner and Gujer (1986)
$Y_{H}$	Yield of *H* on *C*	g(COD) $\text {g(COD)}^{-1}$	0.63	Henze et al. (2006)
$I_{\text {opt}}$	Optimum light intensity	kmol($\hbox {e}^-$) $\hbox {m}^{-2}$ $\hbox {d}^{-1}$	0.01728	Liehr et al. (1990)
$k_{tot}$	Light attenuation coeff	$\hbox {m}^{2}$ $\text {g(COD)}^{-1}$	0.21	Wolf et al. (2007)
$D^L_{S,C}$	Diffusion coeff. of *C* in water	$\hbox {m}^2$ $\hbox {d}^{-1}$	1.04$\cdot 10^{-4}$	Wanner and Gujer (1986)
$D^L_{S,O}$	Diffusion coeff. of $\hbox {O}_2$ in water	$\hbox {m}^2$ $\hbox {d}^{-1}$	2.19$\cdot 10^{-4}$	Wanner and Gujer (1986)
$k_{col}$	Maximum colonization rate of $\Psi _P$	$\hbox {d}^{-1}$	$10^{-3 \ (b)}$	Russo et al. (2024)
$D_{\Psi }$	Diffusion coefficient of $\Psi _P$ in biofilm	$\hbox {m}^2$ $\hbox {d}^{-1}$	$10^{-7 \ (b)}$	This study
$\chi _1$	Phototaxis constant rate	g(COD) $\hbox {m}^{-1}$ $\hbox {d}^{-1}$	$10^{-5 \ (b)}$	This study
$\chi _2$	Phototaxis saturation constant	g(COD) $\hbox {m}^{-3}$	$1^{(b)}$	This study
$\Psi ^*_{P}$	Conc. of planktonic *P* cells at $\Gamma _{B}(t)$	g(COD) $\hbox {m}^{-3}$	1	This study
$S^*_{C}$	Conc. of *C* at $\Gamma _{L}$	g(COD) $\hbox {m}^{-3}$	100	This study
$S^*_{O}$	Conc. of $\hbox {O}_2$ at $\Gamma _{L}$	mol($\hbox {O}_2$) $\hbox {m}^{-3}$	0	This study
$I_0$	Light intensity at $\Gamma _{S}$	kmol $\hbox {m}^{-2}$ $\hbox {d}^{-1}$	$0.01728^{\ (c)}$	This study

(*a*) varied in Sect.  3.3

(*b*) varied in Sect.  3.1

(*c*) varied in Sects.  3.2 and 3.3

Initial and boundary conditions are set as follows. Equation (6) is subject to a homogeneous Dirichlet condition at the biofilm-liquid interface $\Gamma _{B}(t)$, i.e., $p(\textbf{x}, t) = 0$ for $\textbf{x} \in \Gamma _{B}(t)$. This assumption is justified by the much lower viscosity of the bulk liquid compared to that of the biomass, implying that only small pressure gradients are needed in $\Omega \backslash \Omega _B(t)$ for the flow in the bulk liquid to adapt to that of the biomass (Alpkvist and Klapper 2007). At the biofilm-substratum interface, a homogeneous Neumann condition is imposed, reflecting the assumption of an impermeable substratum i.e., $\textbf{n} \cdot \nabla p(\textbf{x}, t) = 0$ for $\textbf{x} \in \Gamma _{S}$. Fixed Dirichlet boundary conditions are prescribed on the top boundary for oxygen and organic carbon: $S_O({\textbf {x}},t) = S^*_O$ and $S_C({\textbf {x}},t) = S^*_C$, for $\textbf{x} \in \Gamma _{L}$, where $S^*_O$ and $S^*_C$ are prescribed concentrations. Specifically, organic carbon was set in the range of values reported in the literature for municipal wastewater, while oxygen was set to zero, considering photosynthetic activity as the only driving force to produce the oxygen necessary for carbon oxidation (Foladori et al. 2018). A Dirichlet boundary condition is prescribed for the planktonic species *P* at the biofilm-liquid interface $\Gamma _{B}(t)$, representing the continuous supply of planktonic cells from the surrounding liquid medium, i.e., $\Psi _P(\textbf{x},t) = \Psi _P^*$ for $\textbf{x} \in \Gamma _{B}(t)$, where $\Psi _P^*$ denotes the prescribed concentration. Null flux conditions for oxygen, carbon and planktonic phototrophs at the biofilm-substratum interface account for the impermeability of the substratum: $\textbf{n} \cdot \nabla S_O = \textbf{n} \cdot \nabla S_C = \textbf{n} \cdot \big ( -D_{\Psi } \nabla \Psi _P+{\Psi _P \textbf{v}} \big ) = 0$, for $\textbf{x} \in \Gamma _{S}$. Continuity of both concentration and flux is enforced for oxygen and carbon at the biofilm-liquid interface $\Gamma _B(t)$. The initial concentrations of oxygen $S_O({\textbf {x}},0)$, carbon $S_C({\textbf {x}},0)$, and planktonic phototrophs $\Psi _P({\textbf {x}},0)$ are set to $S^*_O$, $S^*_C$ and $\Psi ^*_P$, respectively. Furthermore, it is assumed that the biofilm initially consists only of sessile heterotrophs, that is $f_P({\textbf {x}},0) = 0, \ f_H({\textbf {x}},0) = 1, \ {\textbf {x}} \in \Omega _B(t)$. To model a continuous, boundaryless system in the lateral direction and eliminate edge effects, periodic boundary conditions are applied to the lateral boundaries. Finally, the initial position of the biofilm-liquid interface is prescribed as $\Gamma _B(0)=\Gamma _{B,0}$.

All parameter values are provided in Table 1. The diffusion coefficients of substrates in the biofilm $D^B_{S,j}$ are assumed to be 80% of their corresponding values in water $D^L_{S,j}$, following a standard practice in biofilm modeling literature (Wanner and Gujer 1986).

## Numerical Investigations

**Fig. 3:**
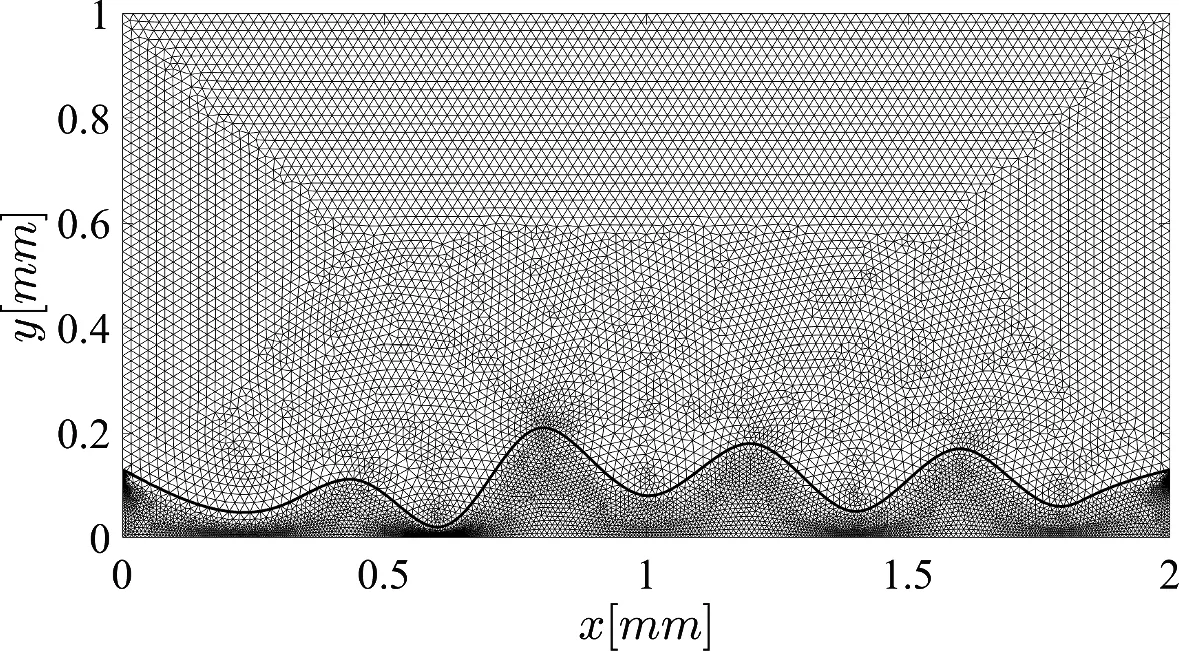
Numerical discretization of the 2D computational domain for simulation purposes (Color figure online)

Numerical simulations were performed using COMSOL Multiphysics (v. 5.4), a software platform based on the finite element method (FEM). The computational domain Ω was defined as a rectangular region of dimensions $W \times L = 2000 \times 1000 \ \mu m$. The initial biofilm-liquid interface was generated by assigning random vertical coordinates y within the interval 20–200 μm to a set of points distributed along the horizontal direction x, and then interpolating these points to obtain a continuous interface. This initial condition is adopted in the studies presented in Sects.  3.1–3.3, while alternative geometries are considered in Sect.  3.4 to assess their influence on biofilm dynamics. To accurately resolve the sharp spatial gradients arising within the biofilm, the region $\Omega _B$ was discretized using a highly refined mesh (see Fig.  3). A very fine mesh was also employed in the liquid region $\Omega \backslash \Omega _B(t)$, although with a lower element density owing to the homogeneous diffusion equations governing this region. The final mesh consisted of 23151 finite elements. During the numerical integration, the equations governing $f_P({{\textbf {x}}},t)$ and $f_H({{\textbf {x}}},t)$ were integrated independently, without explicitly enforcing the closure condition $f_P({{\textbf {x}}},t) + f_H({{\textbf {x}}},t) = 1$. This choice avoids introducing additional algebraic constraints into the integration scheme and allows the dynamical evolution to be determined solely by the governing differential equations. The closure condition was subsequently evaluated and used as an a posteriori measure of numerical accuracy.

### Effect of Random and Phototactic Motility on Planktonic Cells Distribution and Biofilm Ecology

**Fig. 4:**
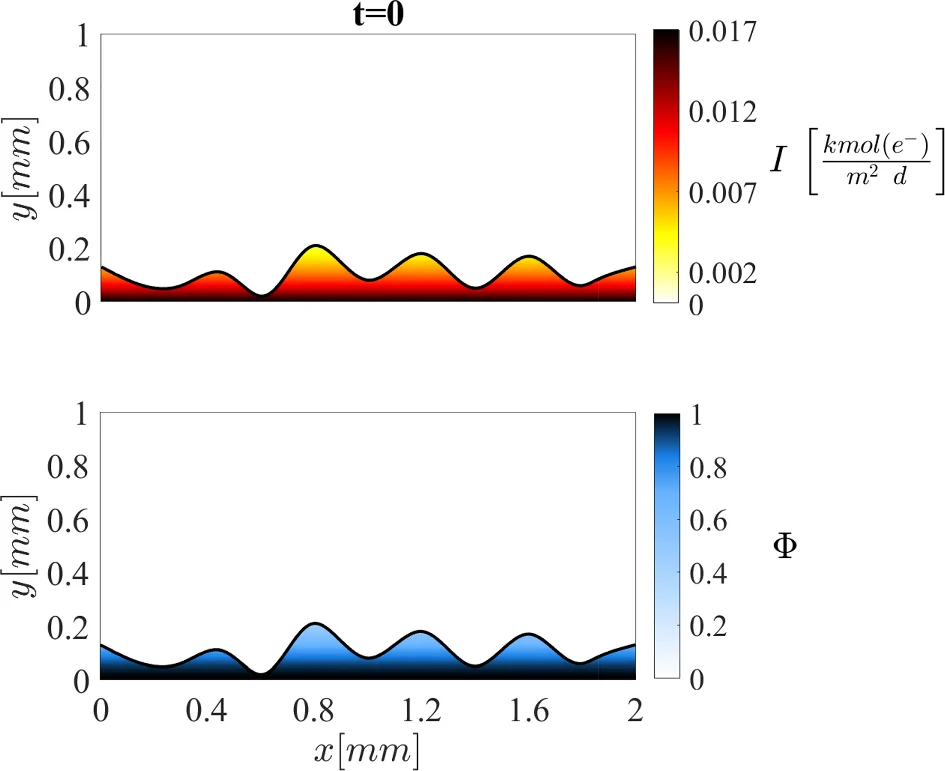
Initial distribution of light intensity *I* and phototactic function Φ within the biofilm domain, in the case of phototactic motility. The light intensity is set at $I_0=I_{\text {opt}} = 0.01728$ kmol ($\hbox {e}^-$) $\hbox {m}^{-2}$ $\hbox {d}^{-1}$ (Color figure online)

**Fig. 5:**
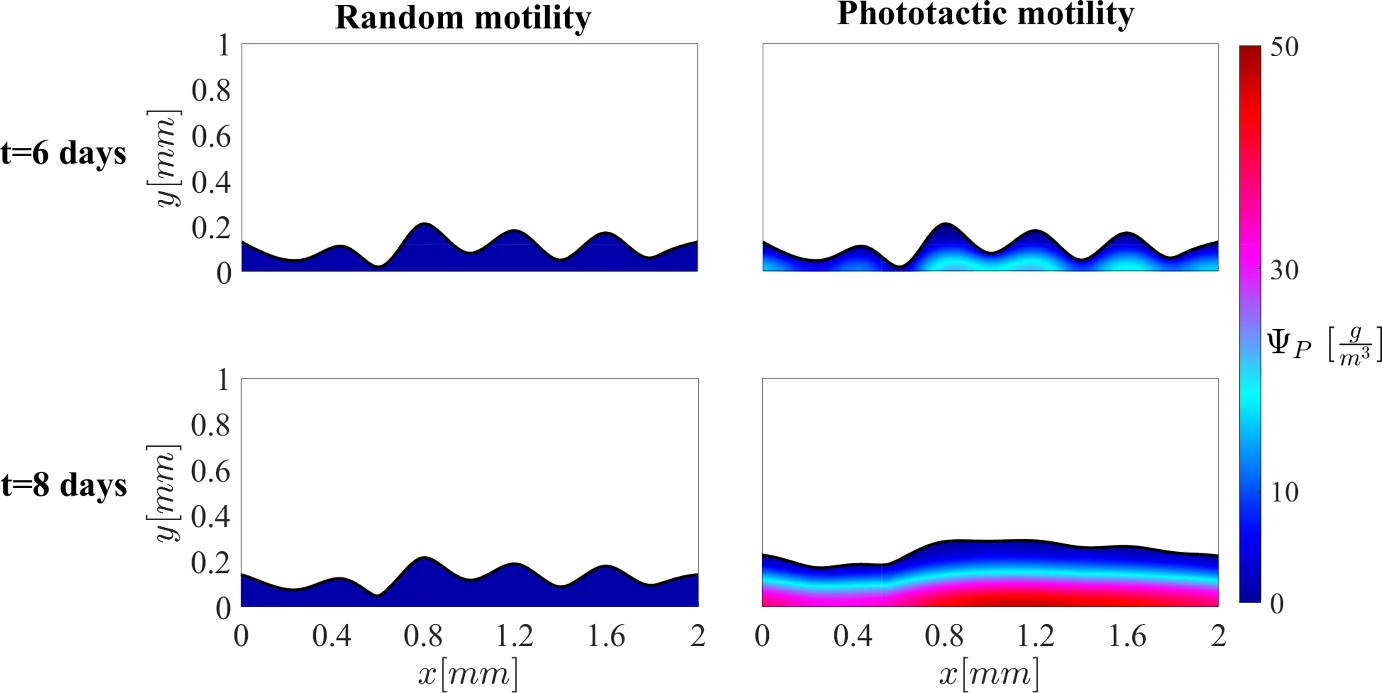
Distribution of planktonic cells within the biofilm in the case of random and phototactic motility at 6 and 8 days. The light intensity is set at $I_0=I_{\text {opt}}=0.01728$ kmol ($\hbox {e}^-$) $\hbox {m}^{-2}$ $\hbox {d}^{-1}$. Random motility: $D_{\Psi } =$ $10^{-7}$ $\hbox {m}^2$ $\hbox {d}^{-1}$, $\chi _1 =$ 0. Phototactic motility: $D_{\Psi } =$ $10^{-7}$ $\hbox {m}^2$ $\hbox {d}^{-1}$, $\chi _1 =$ $10^{-5}$ g(COD) $\hbox {m}^{-1}$ $\hbox {d}^{-1}$ (Color figure online)

To investigate the role of phototaxis in the dynamics of planktonic species and biofilm ecology, this numerical study considers two simulation scenarios: one in which planktonic cells move randomly, without phototaxis ($D_{\Psi } \ne$ 0, $\chi _1 =$ 0), and another in which their movement includes a phototactic component ($D_{\Psi } \ne$ 0, $\chi _1 \ne$ 0). The light intensity provided at the substratum $I_0$ is set to the optimal value $I_{\text {opt}}$. The main variable directly influencing phototactic movement is the phototactic function Φ, which varies within the domain according to light intensity *I*. The behavior of Φ and *I*, shown in Fig.  4, suggests a phototactic movement of planktonic cells toward the substratum. This observation is confirmed by results in Fig.  5, which reports the spatial distribution of planktonic cells within the biofilm domain. When phototaxis is active, planktonic cells exhibit a directed movement toward the illuminated substratum, leading to a marked accumulation of biomass in this region. This contrasts sharply with the case where phototaxis is absent, in which the movement of planktonic cells is purely diffusive. Under such conditions, the planktonic species distribute homogeneously throughout the domain, reaching a concentration close to the fixed boundary value.

**Fig. 6:**
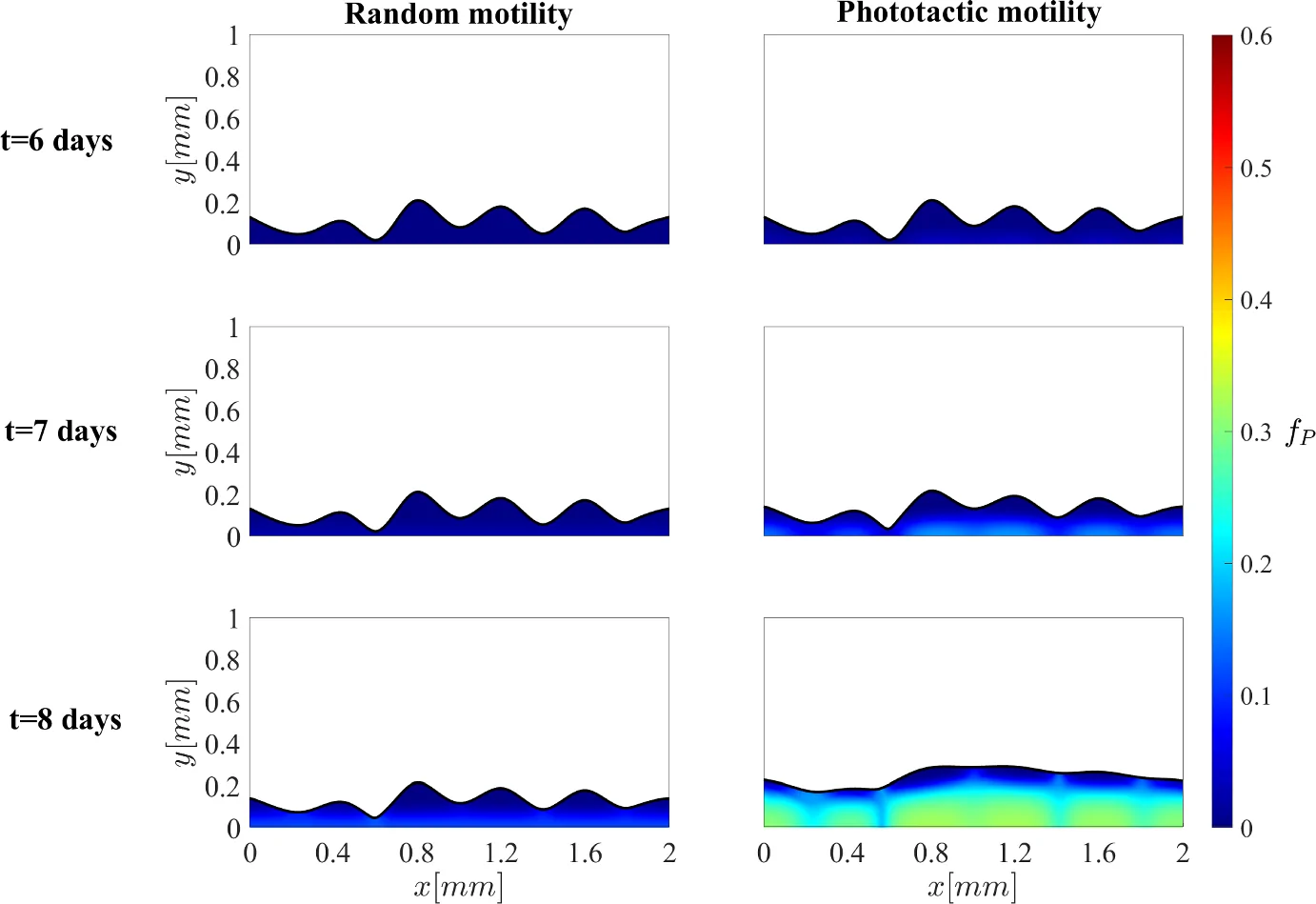
Distribution of sessile phototrophs $f_P$ within the biofilm in the case of random and phototactic motility, at 6, 7 and 8 days. The light intensity is set at $I_0=I_{\text {opt}}=0.01728$ kmol ($\hbox {e}^-$) $\hbox {m}^{-2}$ $\hbox {d}^{-1}$. Random motility: $D_{\Psi } =$ $10^{-7}$ $\hbox {m}^2$ $\hbox {d}^{-1}$, $\chi _1 =$ 0. Phototactic motility: $D_{\Psi } =$ $10^{-7}$ $\hbox {m}^2$ $\hbox {d}^{-1}$, $\chi _1 =$ $10^{-5}$ g(COD) $\hbox {m}^{-1}$ $\hbox {d}^{-1}$ (Color figure online)

**Fig. 7:**
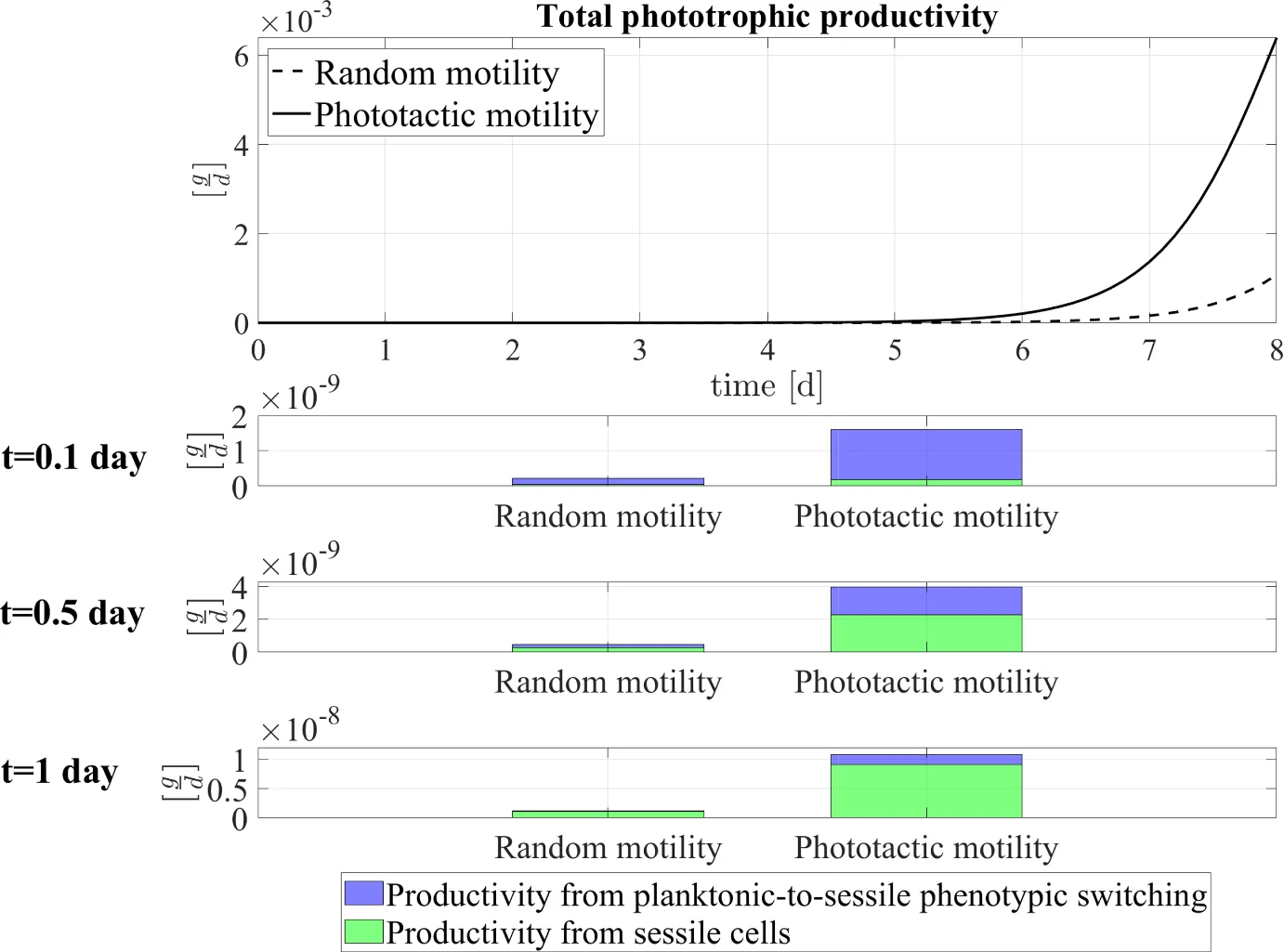
Phototrophic productivity within the biofilm under random and phototactic motility. Top panel: time evolution of the total productivity. Bottom panel: contribution of planktonic-to-sessile switching and growth of pre-existing sessile phototrophs to the total productivity at 0.1, 0.5, and 1 day. The light intensity is set at $I_0=I_{\text {opt}}=0.01728$ kmol ($\hbox {e}^-$) $\hbox {m}^{-2}$ $\hbox {d}^{-1}$. Random motility: $D_{\Psi } =$ $10^{-7}$ $\hbox {m}^2$ $\hbox {d}^{-1}$, $\chi _1 =$ 0. Phototactic motility: $D_{\Psi } =$ $10^{-7}$ $\hbox {m}^2$ $\hbox {d}^{-1}$, $\chi _1 =$ $10^{-5}$ g(COD) $\hbox {m}^{-1}$ $\hbox {d}^{-1}$. Note the different scales on the vertical axes (Color figure online)

**Fig. 8:**
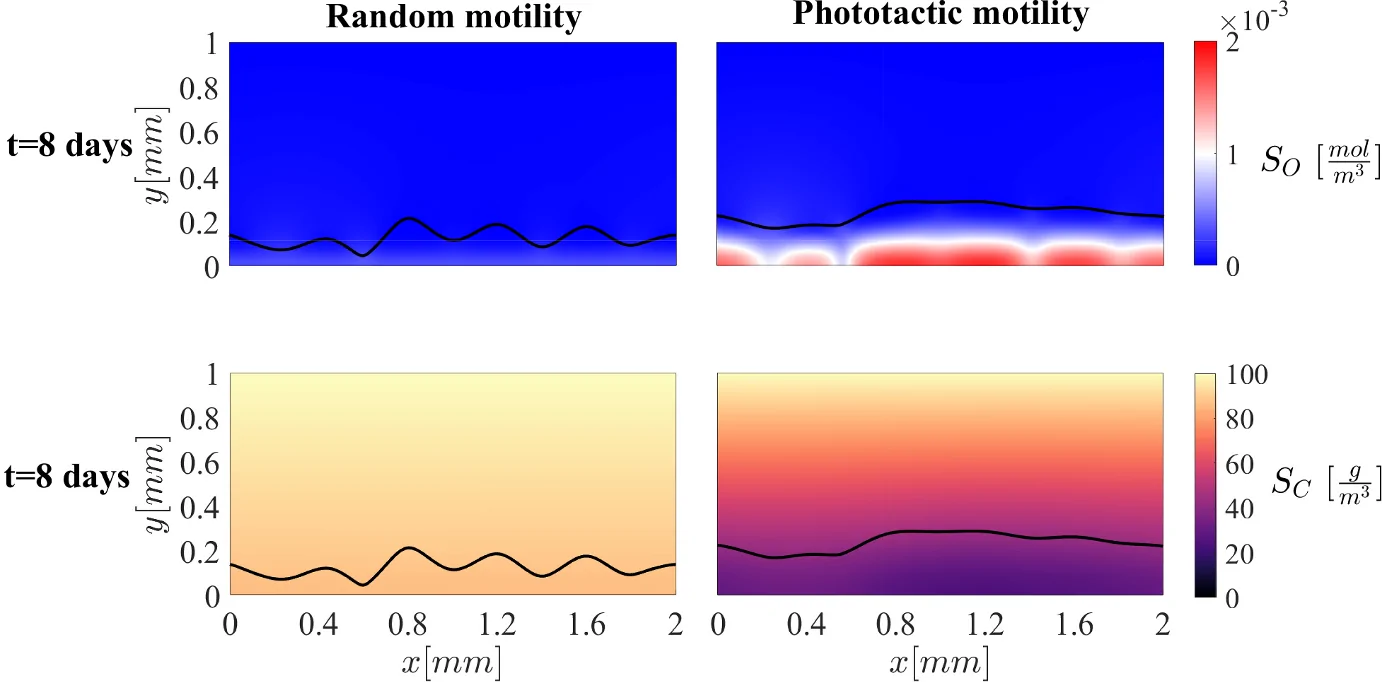
Distribution of oxygen and carbon at 8 days, in the case of random and phototactic motility. The light intensity is set at $I_0=I_{\text {opt}}=0.01728$ kmol ($\hbox {e}^-$) $\hbox {m}^{-2}$ $\hbox {d}^{-1}$. Random motility: $D_{\Psi } =$ $10^{-7}$ $\hbox {m}^2$ $\hbox {d}^{-1}$, $\chi _1 =$ 0. Phototactic motility: $D_{\Psi } =$ $10^{-7}$ $\hbox {m}^2$ $\hbox {d}^{-1}$, $\chi _1 =$ $10^{-5}$ g(COD) $\hbox {m}^{-1}$ $\hbox {d}^{-1}$ (Color figure online)

**Fig. 9:**
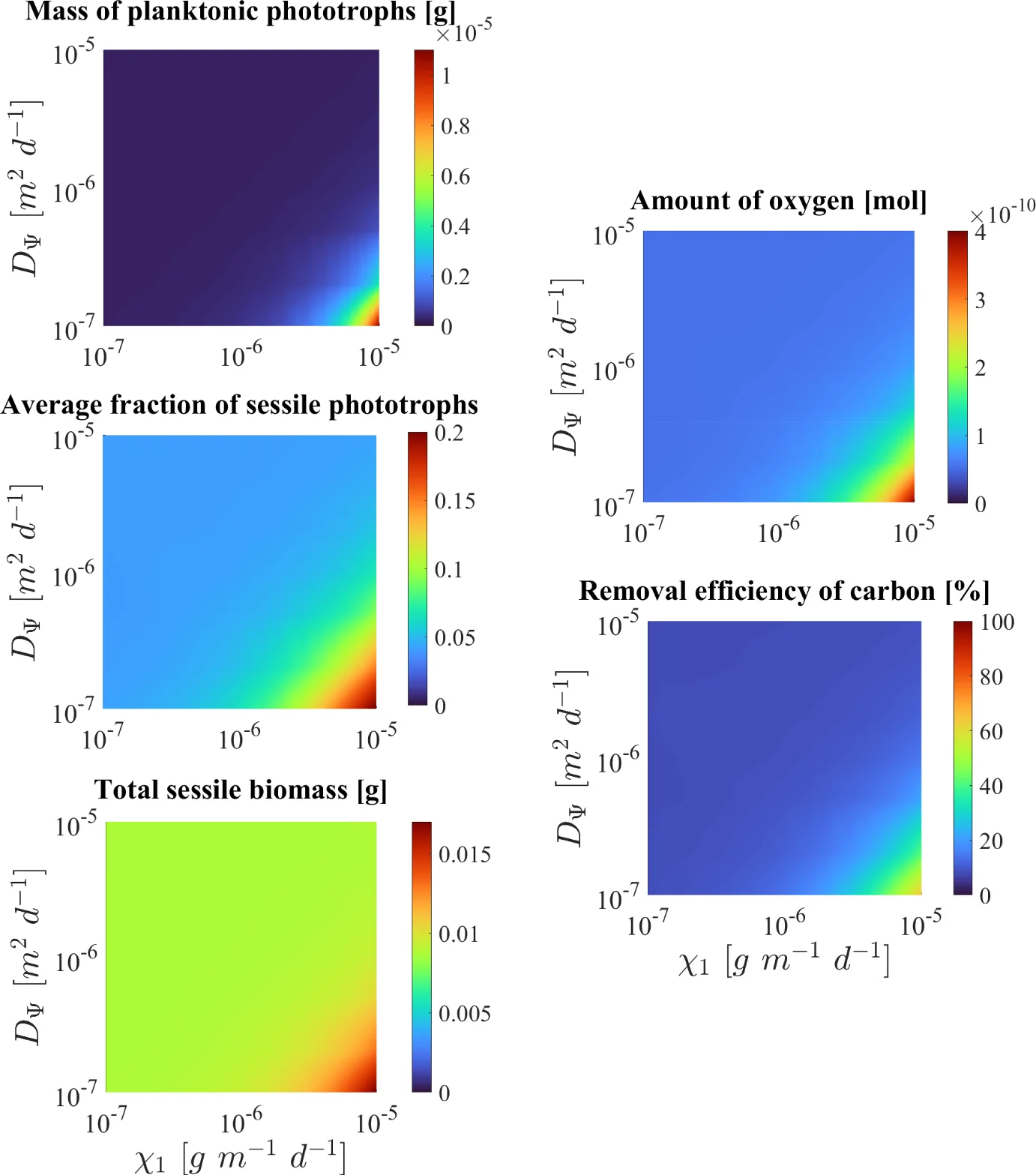
Heatmaps showing the effects of the phototactic sensitivity $\chi _1$ and the random motility coefficient $D_{\Psi }$ on key system indicators: total mass of phototrophic planktonic cells; average fraction of sessile phototrophs; total sessile biomass; amount of oxygen; percentage removal of organic carbon. Warmer colors corresponding to higher values. Logarithmic scales are used for both axes. The light intensity is set at $I_0=I_{\text {opt}} = 0.01728$ kmol ($\hbox {e}^-$) $\hbox {m}^{-2}$ $\hbox {d}^{-1}$. The total sessile biomass serves as a qualitative measure of biofilm growth, being directly proportional to the biofilm volume under the assumption of constant density ρ. All quantities are reported at 8 days (Color figure online)

**Fig. 10:**
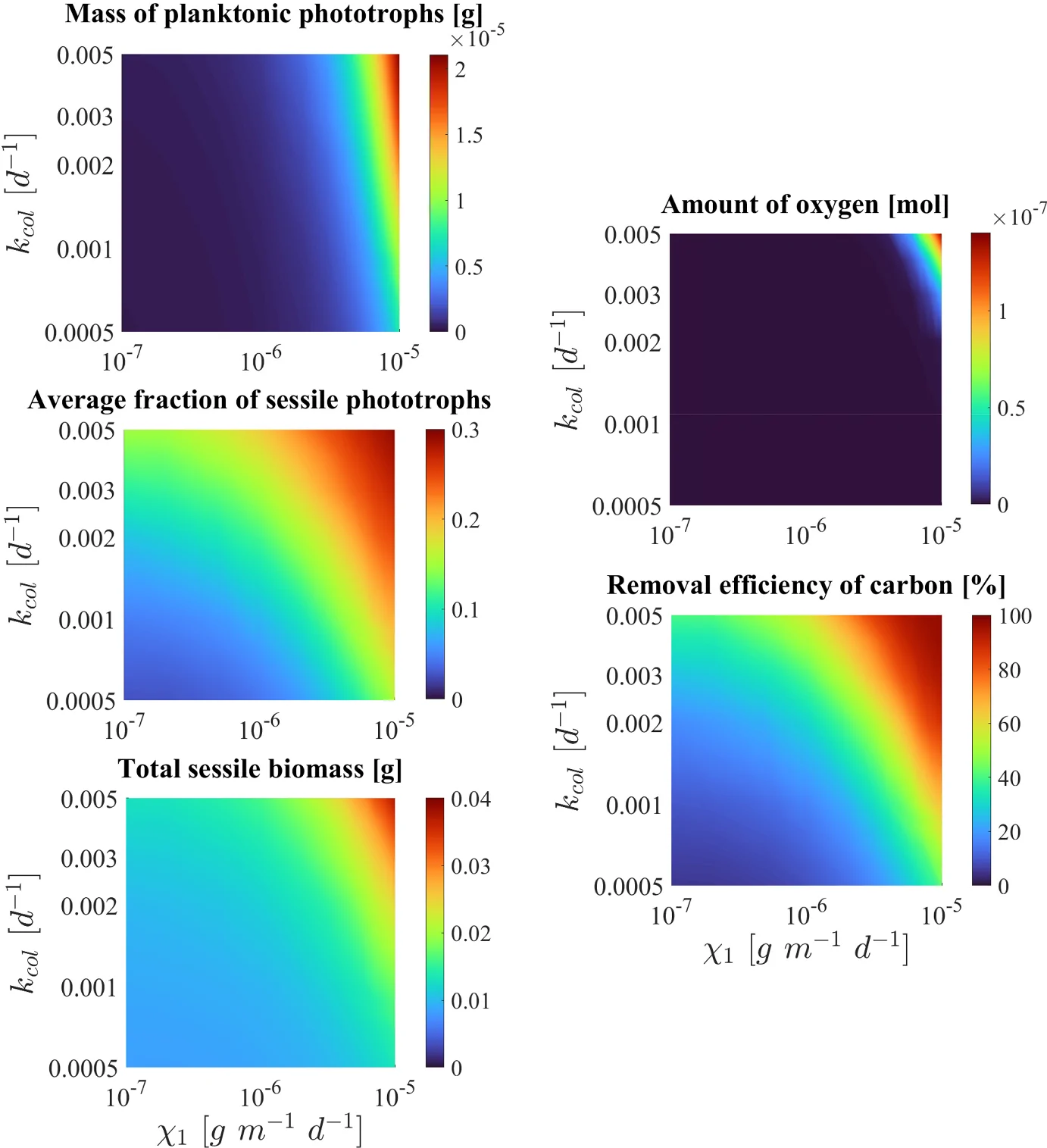
Heatmaps showing the effects of the phototactic sensitivity $\chi _1$ and the planktonic-to-sessile conversion rate $k_{col}$ on key system indicators: total mass of phototrophic planktonic cells; average fraction of sessile phototrophs; total sessile biomass; amount of oxygen; percentage removal of organic carbon. Warmer colors corresponding to higher values. Logarithmic scales are used for both axes. The light intensity is set at $I_0=I_{\text {opt}} = 0.01728$ kmol ($\hbox {e}^-$) $\hbox {m}^{-2}$ $\hbox {d}^{-1}$. The total sessile biomass serves as a qualitative measure of biofilm growth, being directly proportional to the biofilm volume under the assumption of constant density ρ. All quantities are reported at 8 days (Color figure online)

**Fig. 11:**
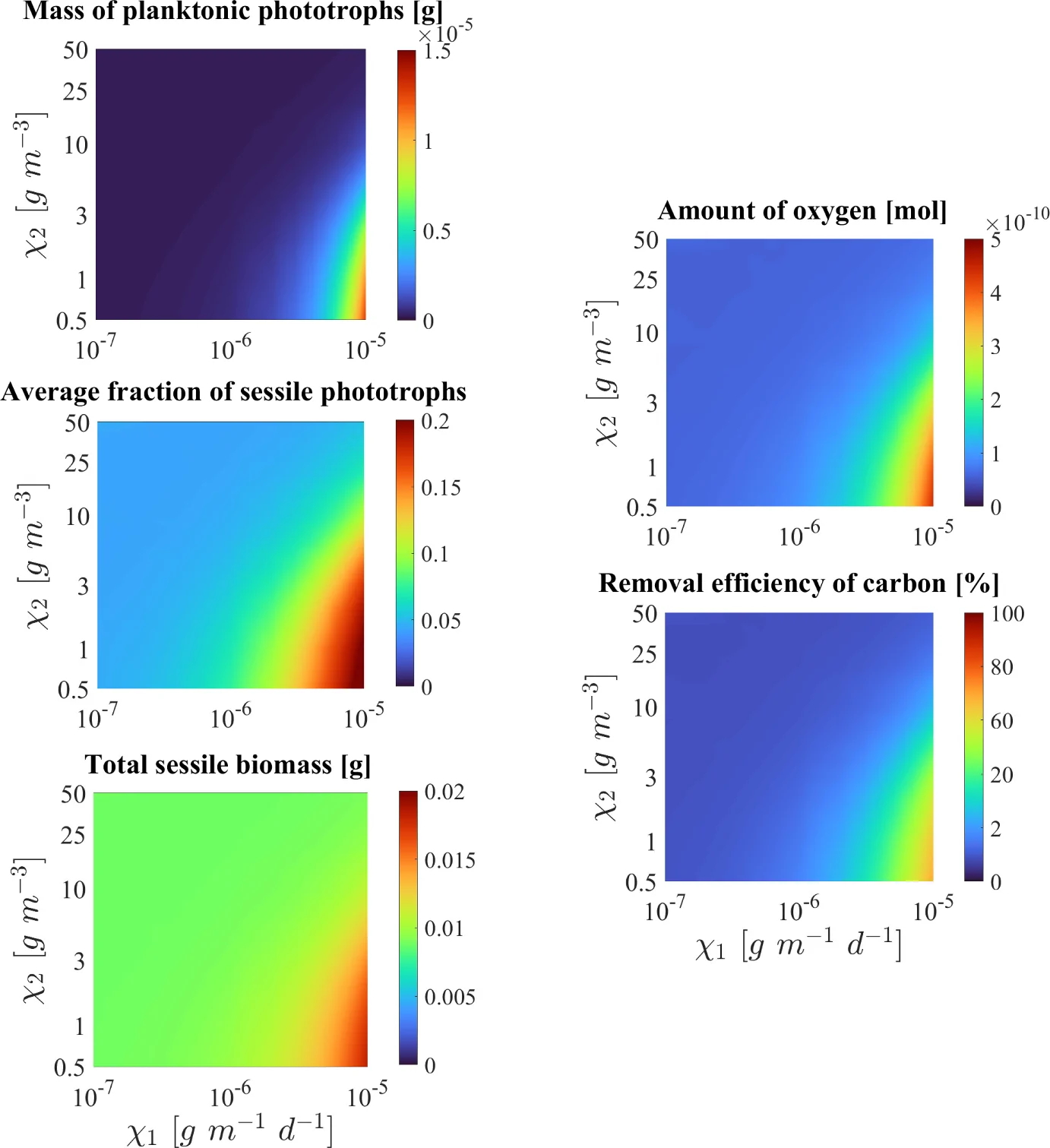
Heatmaps showing the effects of the phototactic sensitivity $\chi _1$ and the chemotactic saturation coefficient $\chi _2$ on key system indicators: total mass of phototrophic planktonic cells; average fraction of sessile phototrophs; total sessile biomass; amount of oxygen; percentage removal of organic carbon. Warmer colors corresponding to higher values. Logarithmic scales are used for both axes. The light intensity is set at $I_0=I_{\text {opt}} = 0.01728$ kmol ($\hbox {e}^-$) $\hbox {m}^{-2}$ $\hbox {d}^{-1}$. The total sessile biomass serves as a qualitative measure of biofilm growth, being directly proportional to the biofilm volume under the assumption of constant density ρ. All quantities are reported at 8 days (Color figure online)

These differences lead to distinct outcomes in biofilm development and composition. Figure  6 depicts the spatial arrangement of sessile phototrophic biomass within the domain. The preferential accumulation of planktonic phototrophs induced by light-driven motility in the inner biofilm regions promotes a rapid and pronounced proliferation of sessile phototrophs, a mechanism that is essentially absent under purely random motility conditions. This mechanism becomes even clearer when examining the phototrophic productivity terms reported in Fig.  7 at different time instants. In particular, the total phototrophic productivity over time (top panel) is computed as the sum of two contributions: (i) the productivity associated with the planktonic-to-sessile phenotypic switching, obtained by integrating $r_P$ over the entire biofilm domain, and (ii) the productivity arising from the growth of the pre-existing sessile biomass, computed by integrating $r_{M,P}$ over the same domain. Both contributions are evaluated under the two motility scenarios. The individual contributions at selected time instants are also reported separately (bottom panel). At early times, sessile growth-induced productivity remains limited due to the initially low abundance of sessile biomass. Under these conditions, the dominant contribution to sessile biomass formation arises from phenotypic switching, which is markedly enhanced in the case of phototactic motility as a consequence of the elevated local concentration of planktonic cells near the illuminated regions. As time progresses, the sessile phototrophic abundance increases substantially, and growth of already established sessile biomass becomes progressively more significant, leading to an exponential increase in overall productivity. This result highlights how phototaxis first accelerates biofilm establishment through enhanced switching and subsequently sustains biomass accumulation through amplified sessile growth.

Notably, the distributions of sessile and planktonic species within the biofilm at times earlier than 6 days are not reported, as they provide limited additional information on biofilm evolution. During this initial phase, the population of sessile phototrophs remains negligible, as shown in Fig.  7, thereby limiting oxygen availability and consequently constrained heterotrophic growth. As a result, biomass production and morphological changes are minimal. Results beyond 8 days are also not presented because this study focuses on the initial establishment and invasion dynamics of phototrophs, when invasion and phototaxis are the dominant processes shaping their spatial distribution. At longer timescales, processes not included in the model, such as detachment and dispersal, may become increasingly relevant and govern the subsequent biofilm evolution.

Figure 8 presents the trends of substrates within the biofilm-liquid domain. The substantial growth of sessile phototrophs observed with phototactic motility significantly impacts the dynamics of oxygen and carbon: phototrophic production leads to intense accumulation of oxygen within the domain, which in turn enhances the metabolic activity of heterotrophic species, leading to an increased consumption of carbon, as shown in the same figure. Conversely, under random motility conditions, the lower abundance of sessile phototrophs leads to reduced oxygen production, thereby limiting heterotrophic activity and resulting in lower carbon consumption. These coupled oxygen–carbon dynamics highlight how phototaxis can actively reshape mass transfer and reaction rates within the system, influencing reactor performance, substrate removal efficiency, and overall process stability.

Furthermore, three separate parametric studies were designed to examine the interactions among the parameters that are most relevant to planktonic-cell dynamics and whose quantitative values are currently unavailable in the literature. Each study explores a two-dimensional parameter space by systematically varying the phototaxis rate $\chi _1$, which directly controls the intensity of phototactic motility, in combination with the other key parameters governing planktonic-cell dynamics: (i) $D_{\Psi }$, representing random cellular motility; (ii) $k_{col}$, regulating the transition from planktonic to sessile state; and (iii) $\chi _2$, which represents the saturation of the directed response induced by volume-filling effects.

The selected system indicators consist of key quantities obtained by integrating the relevant variables over the spatial domain, describing the overall biofilm characteristics and the associated substrate dynamics. These include: (i) the total mass of planktonic phototrophs; (ii) the average fraction of sessile phototrophs within the biofilm, from which the heterotrophic fraction can be directly obtained by complementarity; (iii) the total sessile biomass within the biofilm domain, computed as the sum of phototrophic and heterotrophic biomass, representing overall biofilm growth; (iv) the amount of oxygen produced throughout the domain; and (v) the percentage removal of organic carbon within the biofilm, adopted here as a quantitative indicator of reactor performance. These quantities are displayed in Figs. 9, 10 and 11 as color maps spanning the corresponding parameter spaces, enabling a clear visualization of emerging trends and nonlinear interactions among the governing parameters.

Figure 9 illustrates the combined effect of $\chi _1$ and $D_{\Psi }$. The results clearly demonstrate that both parameters, and especially their relative magnitude, play a crucial role in determining the extent of phototactic accumulation and the subsequent proliferation of phototrophic organisms. At low diffusion levels, phototrophic abundance is highly sensitive to $\chi _1$: strong phototaxis promotes sharp clustering of planktonic cells toward the illuminated region, leading to enhanced planktonic-to-sessile transition and higher sessile concentrations. In contrast, as $D_{\Psi }$ increases, the influence of $\chi _1$ progressively weakens, since random displacement counteracts directional migration. In the latter case, cells remain widely dispersed, which in turn reduces local accumulation and limits phototrophic growth. The variations in biofilm properties are mirrored in the metabolic outputs. Regions characterized by strong phototactic accumulation exhibit higher oxygen production due to increased phototrophic activity. This enhanced oxygen availability supports aerobic heterotrophic degradation, leading to improved organic carbon removal. Conversely, when random motility dominates, the reduced clustering of phototrophs limits oxygen production and moderates carbon removal performance. Notably, these results indicate that the dominant control parameter is not the individual value of $\chi _1$ or $D_{\Psi }$, but rather their ratio. When the total phototrophic biomass is analyzed as a function of $\chi _1 /D_{\Psi }$, an approximately linear trend emerges across the investigated parameter space (data not shown), suggesting that phototrophic growth is primarily governed by the balance between directed phototactic response and random motility. This balance ultimately regulates biofilm development as well as the associated oxygen production and carbon consumption.

Figure 10 reports the outcomes of the second parametric study, where $\chi _1$ is varied together with the conversion rate $k_{col}$. A clear synergistic interaction emerges between directed motility and planktonic-to-sessile transition dynamics. For low values of $k_{col}$, even intense phototaxis does not translate into substantial sessile accumulation, as the limited conversion rate constrains biofilm growth and keeps a significant fraction of cells in the planktonic phase. Conversely, as $k_{col}$ increases, the effect of $\chi _1$ becomes markedly amplified: phototactically driven accumulation near the illuminated boundary is rapidly converted into sessile biomass, leading to a sharp increase in total biofilm mass and in the fraction of phototrophs within the biofilm. At first glance, such rapid conversion would suggest a depletion of planktonic cells in the region of high $k_{col}$ values. However, this interpretation must be considered in light of the overall biofilm expansion. As $k_{col}$ increases, the biofilm domain expands, as confirmed by the increase in total sessile biomass reported in the bottom-left panel of Fig.  10, which, owing to the assumption of constant density ρ, serves as a proxy for biofilm domain growth. Consequently, planktonic cells spread over a larger spatial region, maintaining a substantial overall abundance despite their rapid conversion to the sessile phase. The enhanced phototrophic dominance in the region of high $\chi _1$ and high $k_{col}$ is reflected in the reactor performance metrics. Intensified photosynthetic activity leads to substantial oxygen production, that enhances heterotrophic metabolism, promoting the degradation of organic carbon and ultimately leading to complete removal (up to 100%) at the highest values of $\chi _1$ and $k_{col}$. Note that the region of the parameter space characterized by complete carbon removal also coincides with a sharp increase in oxygen levels. This occurs because, once organic carbon is fully depleted, heterotrophic activity becomes strongly limited and the phototrophic oxygen production is no longer balanced by heterotrophic uptake. Overall, Fig.  10 indicates that the conversion rate acts as a regulatory parameter, controlling the extent to which phototactic accumulation is stabilized into a biofilm system, sustaining both biofilm growth and efficient organic carbon removal.

Figure 11 presents the results of the third parametric study, in which the phototactic sensitivity parameter $\chi _1$ is varied together with the saturation coefficient $\chi _2$. A nonlinear interaction emerges between phototactic strength and saturation effects. For low values of $\chi _2$, the directed response is only weakly limited by crowding, and increasing $\chi _1$ leads to strong phototactic accumulation, that promotes enhanced conversion into sessile biomass and results in a pronounced increase in total biofilm mass, as well as in the relative abundance of phototrophs within the biofilm. As $\chi _2$ increases, however, volume-filling effects progressively constrain directed motility. Even at high $\chi _1$, cellular accumulation becomes less pronounced because crowding limits the effective phototactic migration and the associated downstream effects. These results are directly reflected in oxygen production and carbon removal.

**Fig. 12:**
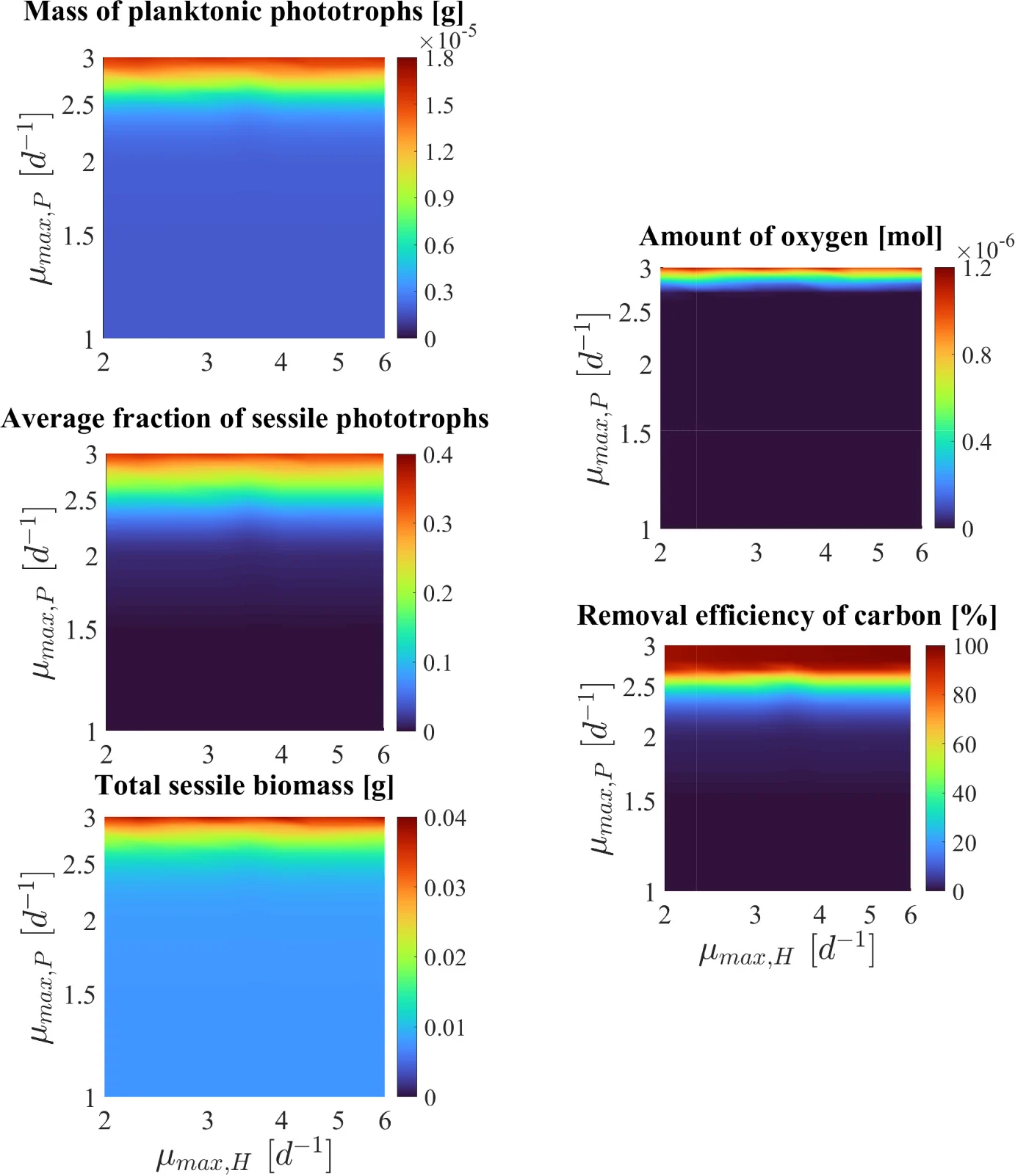
Heatmaps showing the effects of the maximum growth rates of phototrophic and heterotrophic sessile species, $\mu _{max,P}$ and $\mu _{max,H}$, respectively, on key system indicators: total mass of phototrophic planktonic cells; average fraction of sessile phototrophs; total sessile biomass; amount of oxygen; percentage removal of organic carbon. Warmer colors corresponding to higher values. Logarithmic scales are used for both axes. The light intensity is set at $I_0=I_{\text {opt}} = 0.01728$ kmol ($\hbox {e}^-$) $\hbox {m}^{-2}$ $\hbox {d}^{-1}$. The total sessile biomass serves as a qualitative measure of biofilm growth, being directly proportional to the biofilm volume under the assumption of constant density ρ. All quantities are reported at 6 days (Color figure online)

Finally, to quantify the relative influence of species-specific growth kinetics, an additional parametric study was performed by varying the maximum growth rates of phototrophic and heterotrophic sessile species, $\mu _{max,P}$ and $\mu _{max,H}$, respectively. The results are reported in Fig.  12. The effect of these parameters appears to be qualitatively consistent across all selected system indicators: all quantities are strongly affected by variations in $\mu _{max,P}$, whereas the influence of $\mu _{max,H}$ is almost negligible. This result can be understood by considering the distinct roles played by the two microbial populations within the system. Phototrophs act as the invading species and represent the primary driver of biofilm development. Consequently, variations in $\mu _{max,P}$ directly modulate the establishment and subsequent expansion of the phototrophic population, resulting in pronounced changes in biofilm dynamics and associated metabolic processes. Conversely, heterotrophs rely on the oxygen produced by phototrophs to sustain aerobic growth and organic carbon degradation. Therefore, their proliferation is largely regulated by the dynamics of the phototrophic population, and variations in $\mu _{max,H}$ alone have a limited impact when oxygen availability is determined by phototrophic activity. These results highlight that, despite the contribution of both microbial groups to overall biofilm functioning, the system dynamics are mainly governed by the proliferation rate of the invading phototrophic population. It is worth noting that dissolved oxygen exhibits a pronounced increase at very high values of $\mu _{max,P}$. In this region of the parameter space, the organic carbon removal efficiency approaches 100%, causing organic carbon to become the limiting substrate for heterotrophic metabolism. As a result, heterotrophic oxygen consumption is no longer sufficient to balance the oxygen produced by phototrophs, resulting in a marked accumulation of dissolved oxygen within the system.

### Impact of High-Intensity Light on Phototaxis and Biofilm Dynamics

**Fig. 13:**
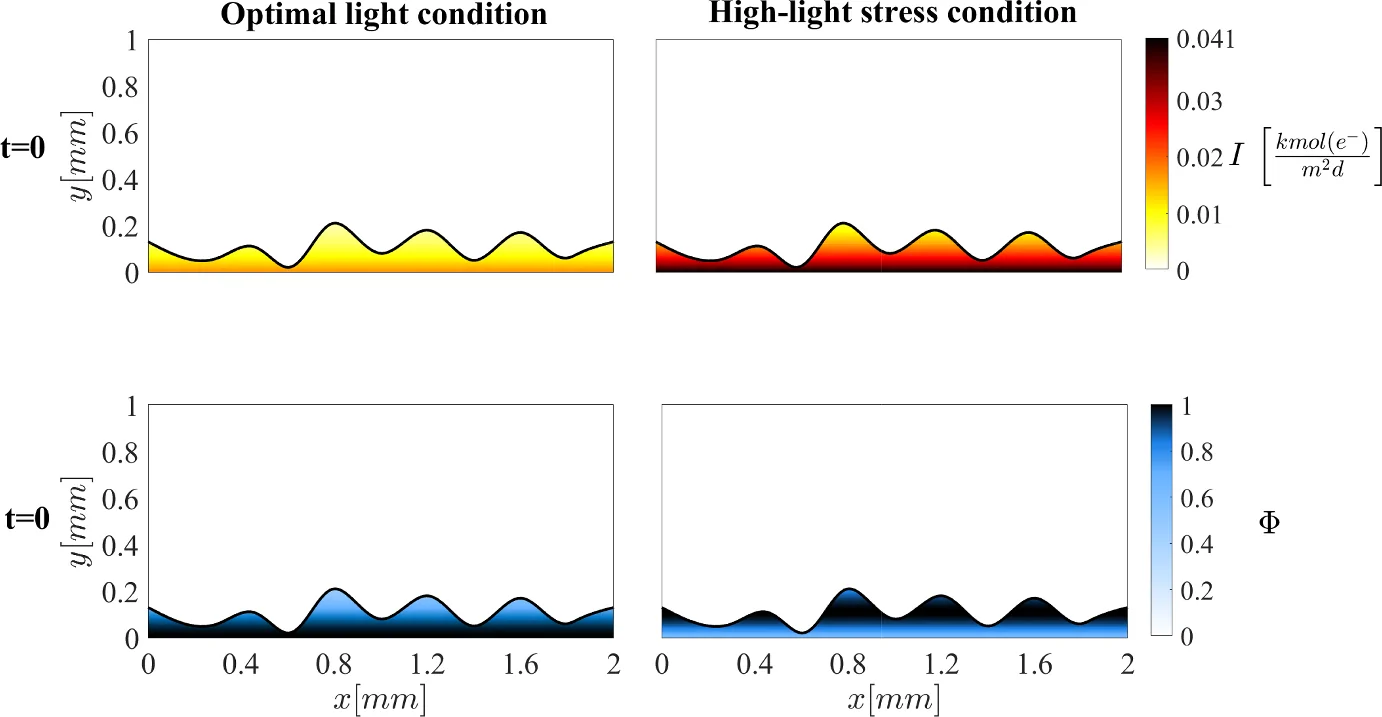
Initial distribution of light intensity *I* and phototactic function Φ within the biofilm under optimal light ($I_0=I_{\text {opt}}=0.01728$ kmol ($\hbox {e}^-$) $\hbox {m}^{-2}$ $\hbox {d}^{-1}$) and high-light stress conditions ($I_0=0.041$ kmol ($\hbox {e}^-$) $\hbox {m}^{-2}$ $\hbox {d}^{-1}$) (Color figure online)

**Fig. 14:**
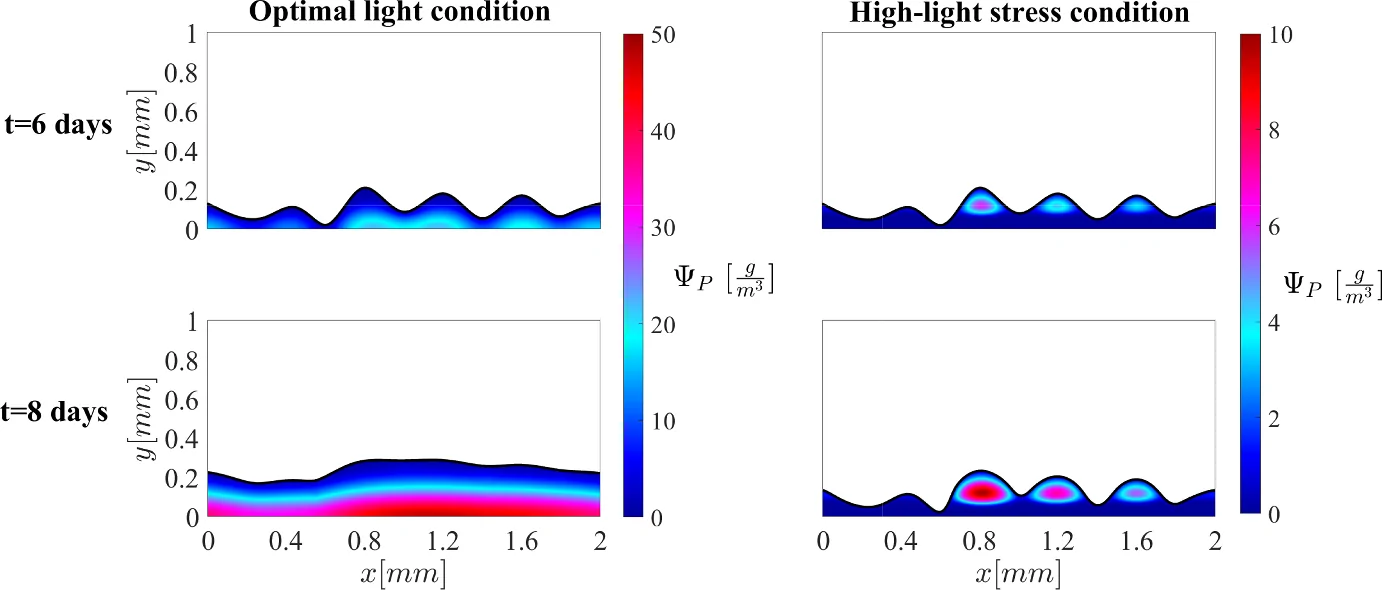
Distribution of planktonic cells within the biofilm under optimal light ($I_0=I_{\text {opt}}=0.01728$ kmol ($\hbox {e}^-$) $\hbox {m}^{-2}$ $\hbox {d}^{-1}$) and high-light stress conditions ($I_0=0.041$ kmol ($\hbox {e}^-$) $\hbox {m}^{-2}$ $\hbox {d}^{-1}$), at 6 and 8 days. $D_{\Psi } =$ $10^{-7}$ $\hbox {m}^2$ $\hbox {d}^{-1}$, $\chi _1 =$ $10^{-5}$ g(COD) $\hbox {m}^{-1}$ $\hbox {d}^{-1}$. Note the different color scales (Color figure online)

**Fig. 15:**
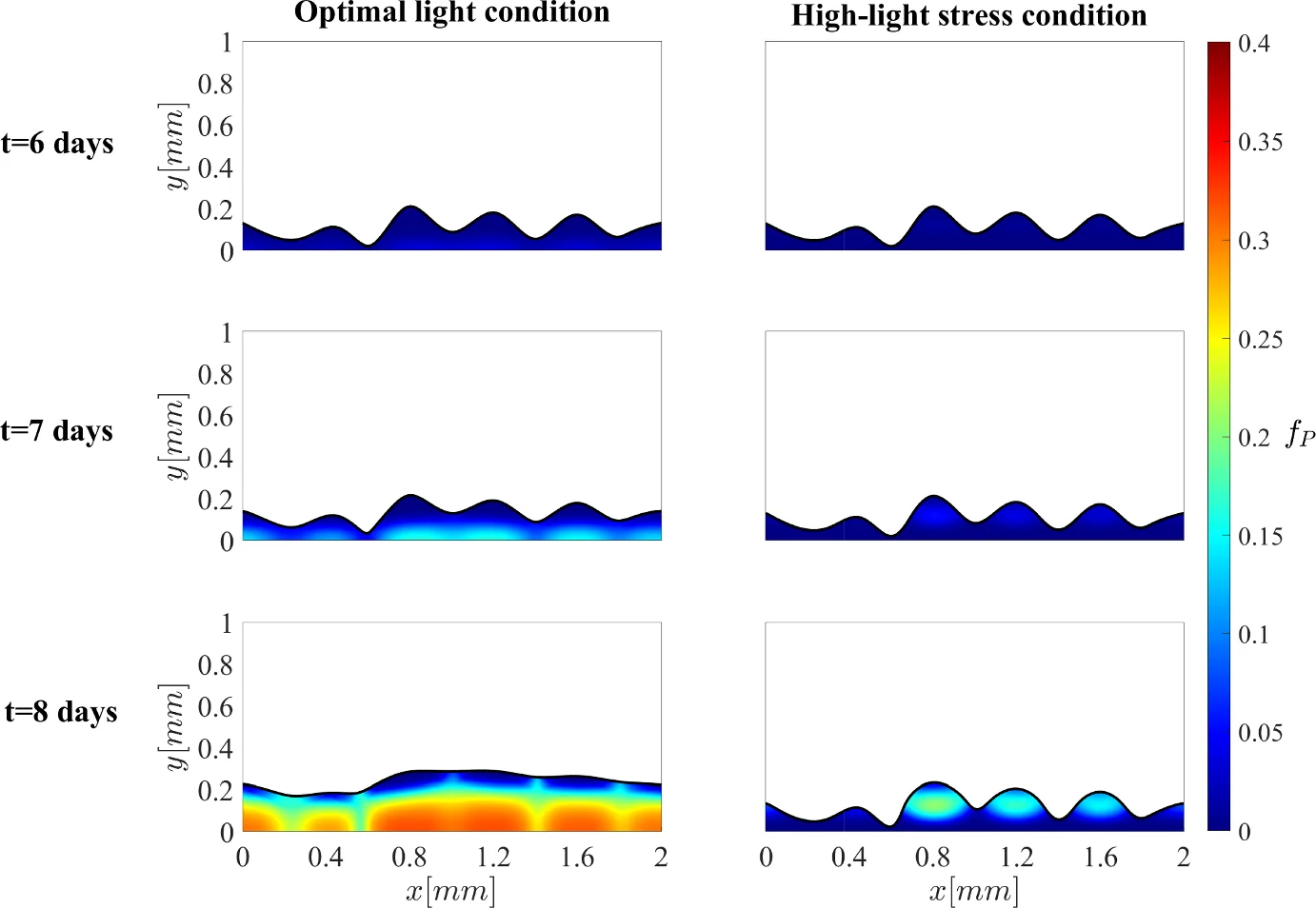
Distribution of sessile phototrophs $f_P$ within the biofilm under optimal light ($I_0=I_{\text {opt}}=0.01728$ kmol ($\hbox {e}^-$) $\hbox {m}^{-2}$ $\hbox {d}^{-1}$) and high-light stress conditions ($I_0=0.041$ kmol ($\hbox {e}^-$) $\hbox {m}^{-2}$ $\hbox {d}^{-1}$), at 6, 7 and 8 days. $D_{\Psi } =$ $10^{-7}$ $\hbox {m}^2$ $\hbox {d}^{-1}$, $\chi _1 =$ $10^{-5}$ g(COD) $\hbox {m}^{-1}$ $\hbox {d}^{-1}$ (Color figure online)

**Fig. 16:**
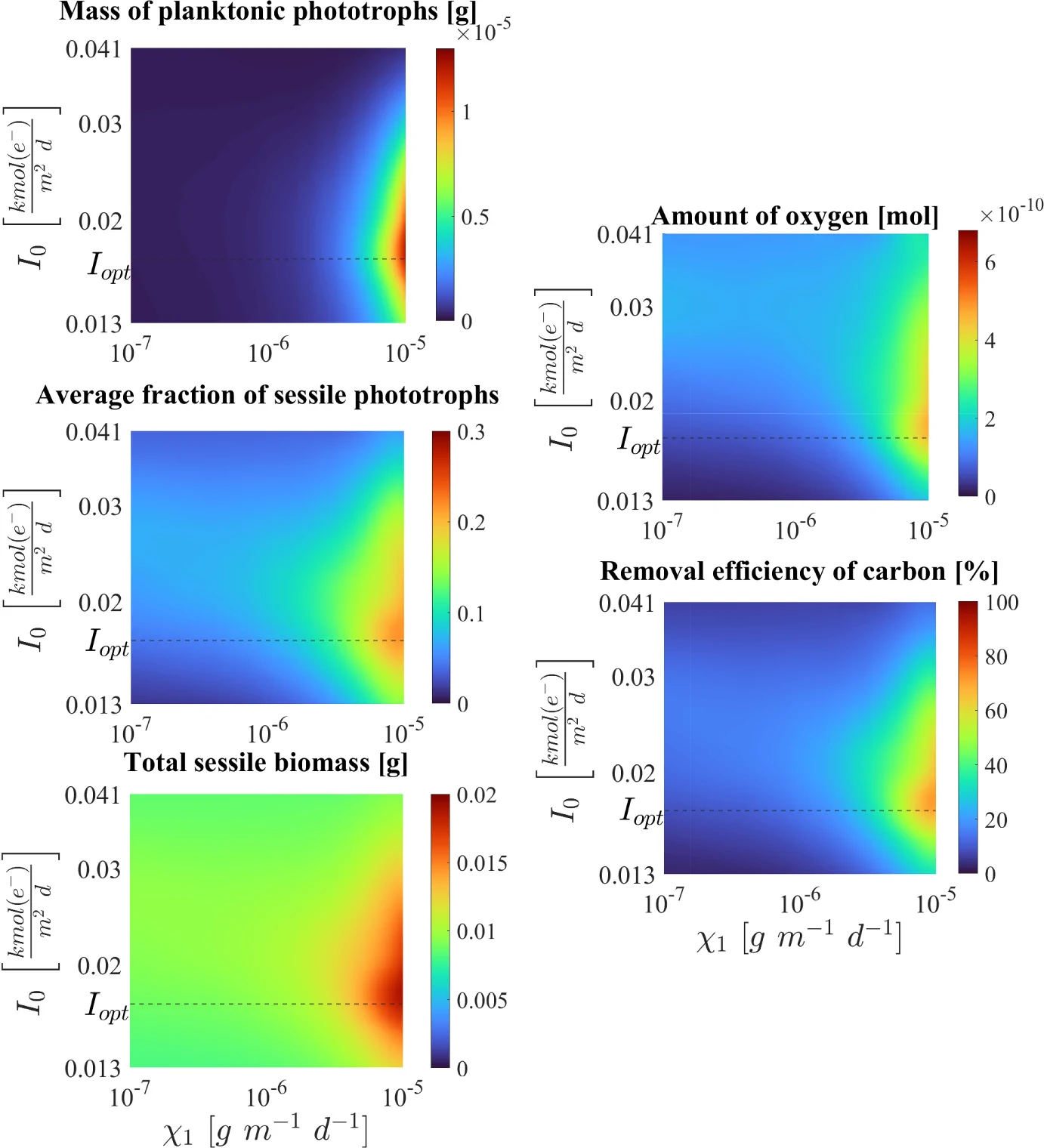
Heatmaps showing the effects of the phototactic sensitivity $\chi _1$ and the incident light intensity $I_0$ on key system indicators: total mass of phototrophic planktonic cells; average fraction of sessile phototrophs; total sessile biomass; amount of oxygen; percentage removal of organic carbon. Warmer colors corresponding to higher values. Logarithmic scales are used for both axes. The total sessile biomass serves as a qualitative measure of biofilm growth, being directly proportional to the biofilm volume under the assumption of constant density ρ. The black dashed line indicates the optimal light intensity $I_{\text {opt}}$. All quantities are reported at 8 days (Color figure online)

Light is not always beneficial for phototrophic species. Under certain high-intensity light conditions, phenomena of photoinhibition can occur, whereby excessive light leads to a decrease in metabolic activity and growth of phototrophs. In these conditions, the phototactic response of phototrophs can shift, resulting in negative phototaxis—where cells move away from the light source rather than toward it. This change in behavior is critical to understanding biofilm dynamics under light stress conditions.

In this numerical study, we examine the effects of light stress conditions on phototaxis and biofilm dynamics by comparing the outcomes under optimal light intensity ($I_0=I_{\text {opt}}$) and light stress conditions, where the light intensity exceeds the optimal value ($I_0=0.041$ kmol ($\hbox {e}^-$) $\hbox {m}^{-2}$ $\hbox {d}^{-1}$). The results are presented through three figures (Figs.  13, 14 and 15), each illustrating a different aspect of biofilm dynamics.

The distribution of light intensity and the corresponding phototactic function within the biofilm domain are shown in Fig.  13, under both light conditions. Under light stress conditions, the light intensity near the substratum exceeds the optimal level for phototrophic activity, leading to reduced values of the phototactic response in these overexposed regions. However, as light penetrates the biofilm, it is gradually attenuated by the upper layers. This attenuation causes the phototactic niche to shift upward—from the region adjacent to the substratum to more superficial layers of the biofilm—where light levels become more favorable for phototaxis. As a result, the phototactic function no longer exhibits a monotonically decreasing trend along the y-axis. Instead, as one moves away from the substratum, the function first increases and then decreases, reaching its maximum value at the depth where $I({\textbf {x}},t)=I_{\text {opt}}$.

As a consequence of the changes in the light intensity profile and the phototactic function, the distribution of planktonic cells within the biofilm undergoes notable alterations, as shown in Fig. 14. Planktonic cells penetrate the biofilm from the surrounding environment, without reaching the deepest layers; instead, they settle at optimal depths for their growth. Similarly, planktonic cells initially located near the substratum move away from it. The result is the formation of distinct pockets of planktonic cells within the biofilm, alimented both by the inner layers, where light intensity is excessive, and by the outer layers, where light is reduced.

Figure 15 illustrates the distribution of sessile phototrophs within the biofilm under both light conditions. Under light stress intensity, the distribution of planktonic cells results in a reduced and localized growth of phototrophic sessile biomass compared to optimal light conditions. As a consequence, the overall lower growth rate leads to a limited biofilm development.

The combined effects of phototactic sensitivity and incident light intensity were also investigated across the ($\chi _1,I_0$) parameter space. In this context, the incident light intensity is the primary operating parameter governing light availability within the biofilm domain, and indirectly regulates planktonic-cell dynamics and, consequently, phototactic response. The resulting system responses are reported in Fig. 16, highlighting the non-monotonic influence of light intensity on biofilm development. When $I_0$ is close to the optimal light level, phototrophic activity is maximized and phototactic migration is effective, leading to increased accumulation of phototrophs and enhanced conversion into sessile biomass. In contrast, when the incident light intensity becomes excessively high, negative phototaxis and photoinhibition reduce cellular accumulation and limit biomass growth, resulting in lower phototrophic biomass despite abundant light. However, these effects are strongly modulated by phototactic sensitivity. At low $\chi _1$, directed motility is weak, and variations in light intensity across the entire $I_0$ interval produce only limited changes in biofilm dynamics. As $\chi _1$ increases, phototactic response becomes more pronounced, amplifying the system’s sensitivity to light intensity: small changes in $I_0$ lead to substantial shifts in the spatial accumulation of planktonic phototrophs, which in turn enhances their transition to the sessile phase and strongly influences the overall biofilm activity. Accordingly, oxygen levels increase in regions of higher phototrophic activity, stimulating aerobic heterotrophic metabolism and improving organic carbon removal efficiency.

**Fig. 17:**
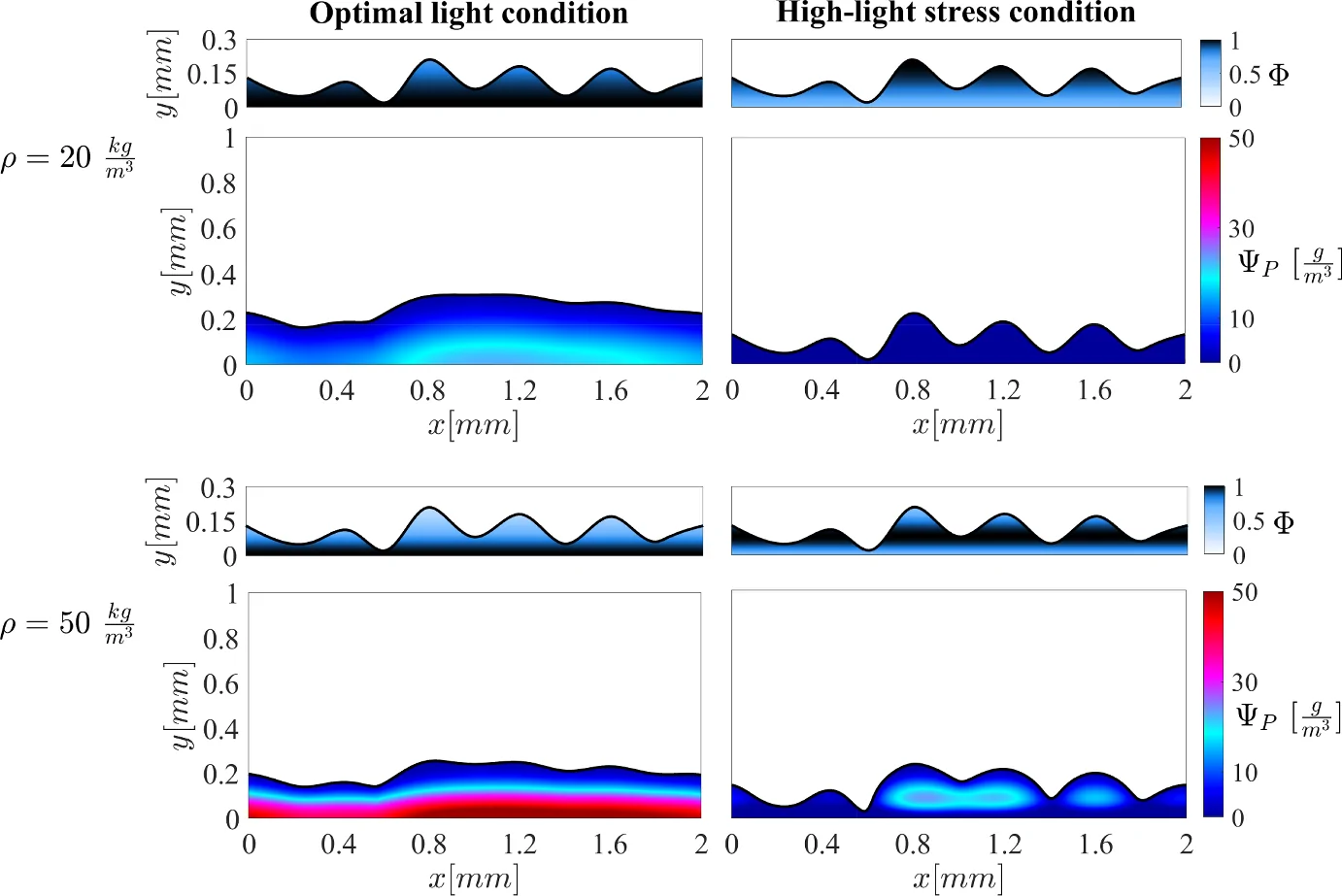
Distribution of phototactic function Φ and planktonic cells $\Psi _P$ within the biofilm at 8 days under optimal light ($I_0=I_{\text {opt}}=0.01728$ kmol ($\hbox {e}^-$) $\hbox {m}^{-2}$ $\hbox {d}^{-1}$) and high-light stress conditions ($I_0=0.041$ kmol ($\hbox {e}^-$) $\hbox {m}^{-2}$ $\hbox {d}^{-1}$), for different biofilm density values. $D_{\Psi } =$ $10^{-7}$ $\hbox {m}^2$ $\hbox {d}^{-1}$, $\chi _1 =$ $10^{-5}$ g(COD) $\hbox {m}^{-1}$ $\hbox {d}^{-1}.$ (Color figure online)

**Fig. 18:**
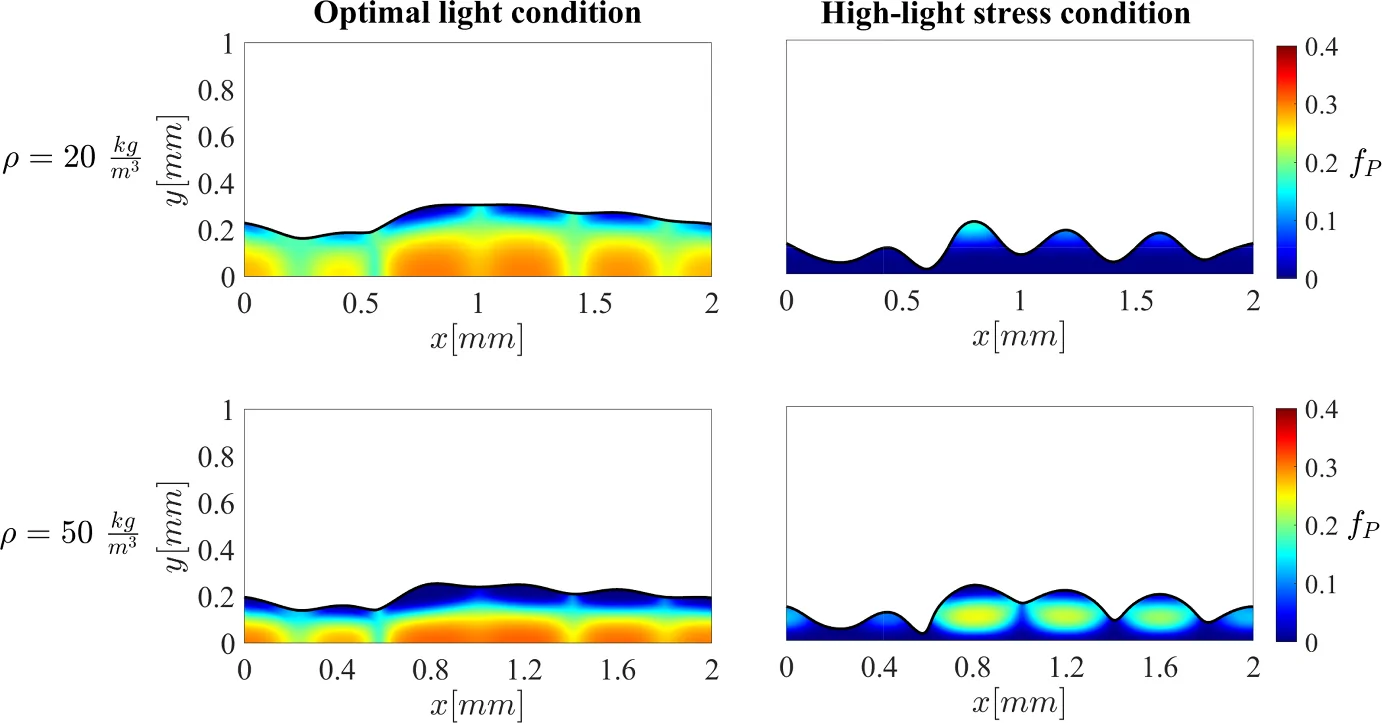
Distribution of sessile phototrophs $f_P$ within the biofilm at 8 days under optimal light ($I_0=I_{\text {opt}}=0.01728$ kmol ($\hbox {e}^-$) $\hbox {m}^{-2}$ $\hbox {d}^{-1}$) and high-light stress conditions ($I_0=0.041$ kmol ($\hbox {e}^-$) $\hbox {m}^{-2}$ $\hbox {d}^{-1}$), for different biofilm density values. $D_{\Psi } =$ $10^{-7}$ $\hbox {m}^2$ $\hbox {d}^{-1}$, $\chi _1 =$ $10^{-5}$ g(COD) $\hbox {m}^{-1}$ $\hbox {d}^{-1}$ (Color figure online)

**Fig. 19:**
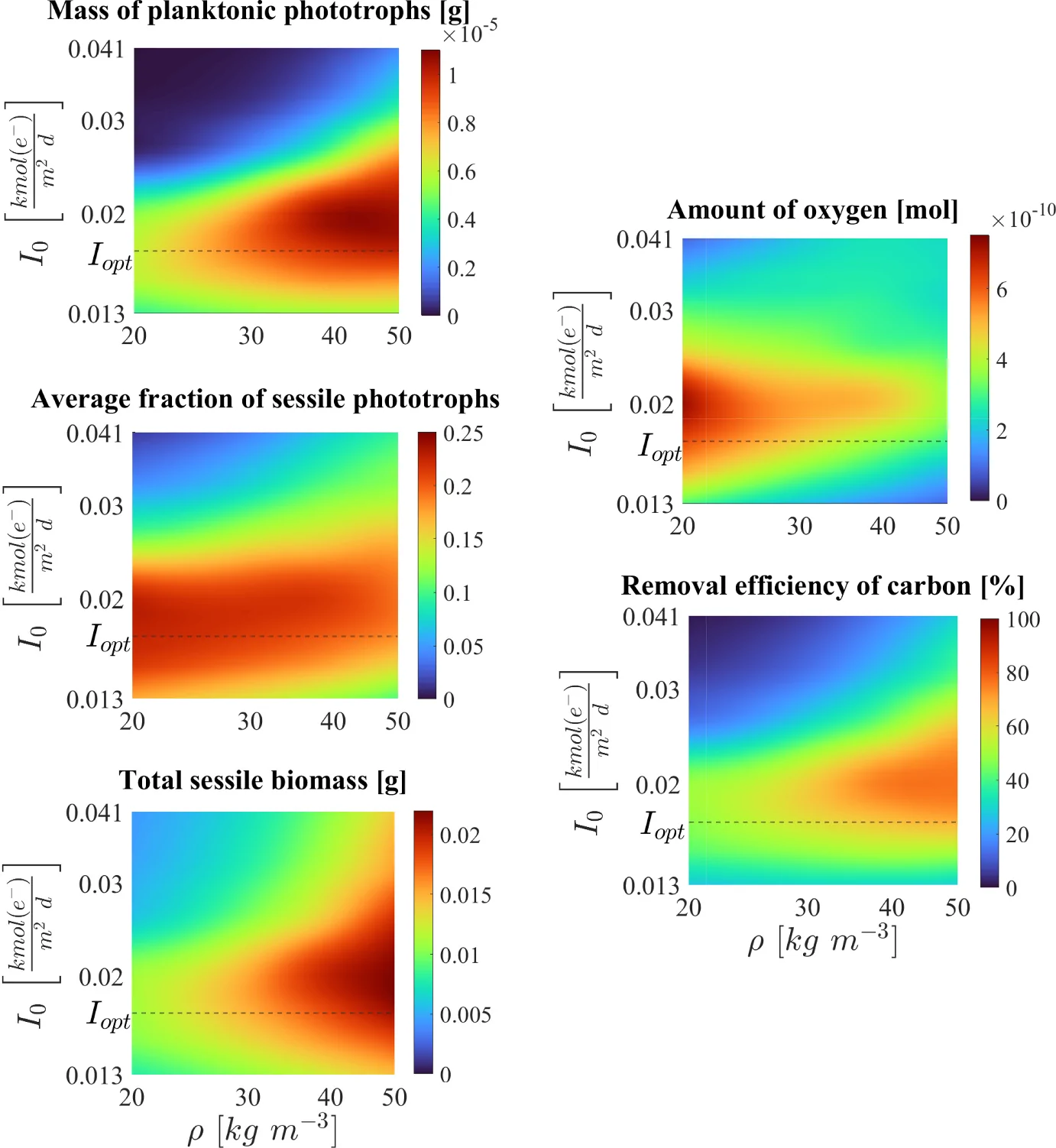
Heatmaps showing the effects of the biofilm density ρ and the incident light intensity $I_0$ on key system indicators: total mass of phototrophic planktonic cells; average fraction of sessile phototrophs; total sessile biomass; amount of oxygen; percentage removal of organic carbon. Warmer colors corresponding to higher values. Logarithmic scales are used for both axes. The total sessile biomass serves as a qualitative measure of biofilm growth, being directly proportional to the biofilm volume under the assumption of constant density ρ. The black dashed line indicates the optimal light intensity $I_{\text {opt}}$. All quantities are reported at 8 days (Color figure online)

### Influence of Biofilm Density on Phototaxis and Light Distribution

The findings outlined in the previous sections underscore the critical influence of light conditions on cell movement and microbial ecosystem development. However, in addition to external illumination, the biofilm’s intrinsic properties—particularly its scattering and absorption characteristics—play a key role in regulating light attenuation within the system. These factors determine the extent of light attenuation and its spatial distribution, ultimately shaping phototactic responses and biofilm dynamics. To further investigate this aspect, we introduce a numerical study exploring the effects of biofilm density on phototaxis. This study is conducted under the two different illumination configurations previously discussed—optimal conditions and high-light stress—allowing for an assessment of how biofilm characteristics modulate phototactic behavior across different environmental conditions.

We conducted four numerical simulations, combining the two light conditions with two different biofilm density values. Figures  17 and 18 illustrate the trends in the phototactic function and the spatial distribution of planktonic and sessile phototrophic species within the domain. Importantly, variations in biofilm density affect the system through multiple and interconnected mechanisms, simultaneously modifying light penetration, phototactic migration, and metabolic activity.

In denser biofilms, stronger light attenuation produces steeper gradients of light intensity, which enhance the directed migration of phototrophic cells toward regions where light conditions remain favorable. As a consequence, planktonic cells accumulate within thinner optimal-growth layers, where their local concentration becomes higher. Although this concentration effect may locally promote biofilm development, it must be considered together with the impact of increased light attenuation on the overall metabolic activity of the system. Under optimal light conditions, higher biofilm density and stronger light attenuation reduce the extent of the biofilm region exposed to ideal light intensities. The resulting limitation in metabolically active volume outweighs the effect of the increased local concentration of planktonic cells, ultimately leading to overall reduced biomass growth, as reflected by a smaller biofilm thickness. In contrast, under high-light stress conditions, stronger light attenuation shifts the light levels closer to the optimal range for phototrophic activity. In this case, the positive effect of reduced photoinhibition combines with the enhanced phototactic accumulation, ultimately promoting a larger biomass growth and a greater biofilm expansion. In other words, increased biofilm density can act as a protective shield under light-stress conditions, attenuating excessive radiation and reducing the exposure of biofilm layers to photoinhibitory intensities.

The interplay between biofilm density and light conditions is further examined in Fig.  19, which shows the system response across the ($\rho ,I_0$) parameter space. Biofilm density plays a key role in modulating light attenuation within the biofilm domain, while the incident light intensity represents the primary external driver of light availability in the system. Their combined effect critically determines the spatial distribution of light and, consequently, governs the resulting planktonic-cell dynamics. The results confirm the intricate coupling between light attenuation, phototactic migration, and metabolic activity, showing how variations in illumination and biofilm density jointly regulate both microbial distribution and overall system performance.

For values of $I_0$ close to optimal light conditions, phototaxis peaks and the abundance of planktonic phototrophs in the system reaches its highest levels. Interestingly, as ρ increases, the range of incident light intensities that support high planktonic accumulation broadens, extending even into light-stress conditions, whose negative effects are mitigated by the increased ρ. For the same reason, the region of the parameter space where the average fraction of phototrophs is maximized shifts slightly toward higher light intensities as ρ increases. The total sessile biomass also increases with ρ. Indeed, although previous analyses indicate that higher biofilm density may, depending on the illumination regime, reduce the overall biofilm expansion, the amount of biomass per unit volume becomes larger, resulting in a higher total sessile mass. Oxygen dynamics are governed by the balance between phototrophic production and heterotrophic consumption. Consequently, the total mass of oxygen in the system reflects the relative abundance of the two microbial groups. In contrast, the dynamics of organic carbon depend exclusively on heterotrophic uptake; therefore, the carbon removal efficiency primarily follows the overall biomass present in the system, increasing in regions of the parameter space where biomass growth is higher.

### Impact of Initial Biofilm Geometry

All previous simulations were performed by considering a specific inoculation on the substratum: a continuous biofilm domain along *x*, whose interface with the liquid was generated by interpolating randomly distributed points along the *y*-direction. However, biofilm geometry can strongly affect the nutrient transport and light attenuation patterns, thereby shaping the overall biofilm ecology.

**Fig. 20:**
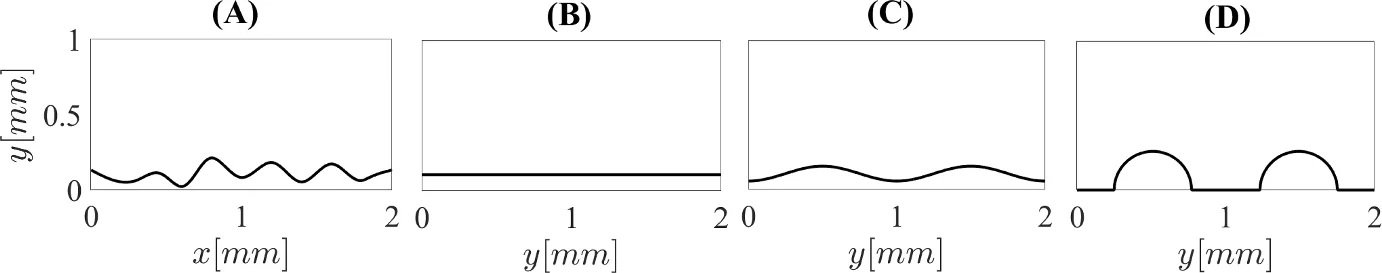
Initial biofilm geometries considered: **A** Reference irregular geometry; **B** Continuous biofilm layer with a flat biofilm-liquid interface and uniform thickness $y=105 \ \mu m$; **C** Continuous biofilm layer with a periodic biofilm-liquid interface defined by $y(x) =105 + 5 \sin (\frac{3\pi }{2}+ 0.002 \pi x)$, where *x* and *y* are expressed in μm; **D** Two disconnected semicircular biofilm colonies of radius $260 \ \mu m$. All geometries have the same total biofilm volume (Color figure online)

For this reason, we assessed the effects of the initial biofilm geometry on the system dynamics by testing different biofilm domains while keeping the total biofilm volume constant. The geometries considered are reported in Fig.  20. The reference irregular geometry, hereafter denoted as geometry (A), was compared with the following alternative configurations: (B) a continuous biofilm layer with a flat biofilm-liquid interface and uniform thickness $y=105 \ \mu m$; (C) a continuous biofilm layer with a periodic biofilm-liquid interface defined by $y(x) =105 + 5 \sin (\frac{3\pi }{2}+ 0.002 \pi x)$, where *x* and *y* are expressed in μm; (D) two disconnected semicircular biofilm colonies of radius $260 \ \mu m$. This set of geometries was selected to represent progressively different interface topologies and spatial distributions of biomass, enabling the evaluation of the influence of the initial biofilm shape independently of the total biomass content.

**Fig. 21:**
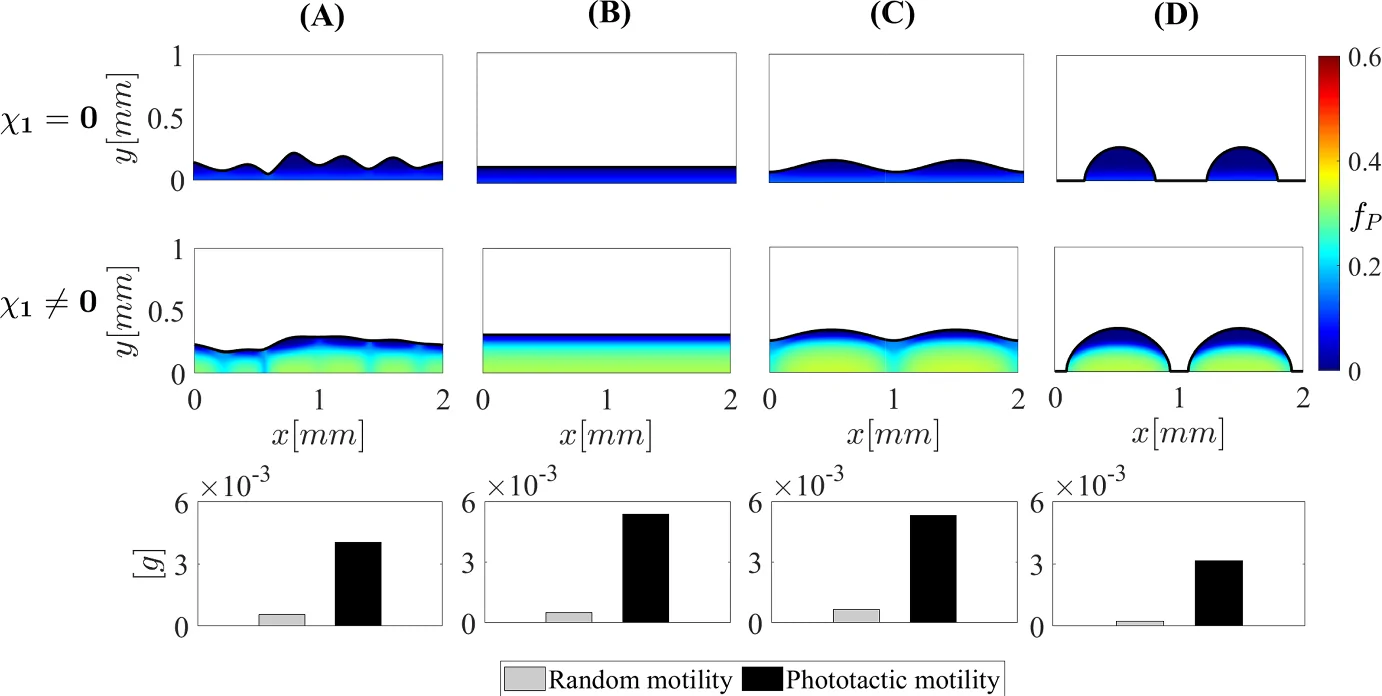
Distribution and total mass of sessile phototrophs $f_P$ within the biofilm under random ($\chi _1 =$ 0) and phototactic ($\chi _1 \ne$ 0) motility, at 8 days, for different initial biofilm geometries. The light intensity is set at $I_0=I_{\text {opt}}=0.01728$ kmol ($\hbox {e}^-$) $\hbox {m}^{-2}$ $\hbox {d}^{-1}$. Random motility: $D_{\Psi } =$ $10^{-7}$ $\hbox {m}^2$ $\hbox {d}^{-1}$, $\chi _1 =$ 0. Phototactic motility: $D_{\Psi } =$ $10^{-7}$ $\hbox {m}^2$ $\hbox {d}^{-1}$, $\chi _1 =$ $10^{-5}$ g(COD) $\hbox {m}^{-1}$ $\hbox {d}^{-1}$ (Color figure online)

**Fig. 22:**
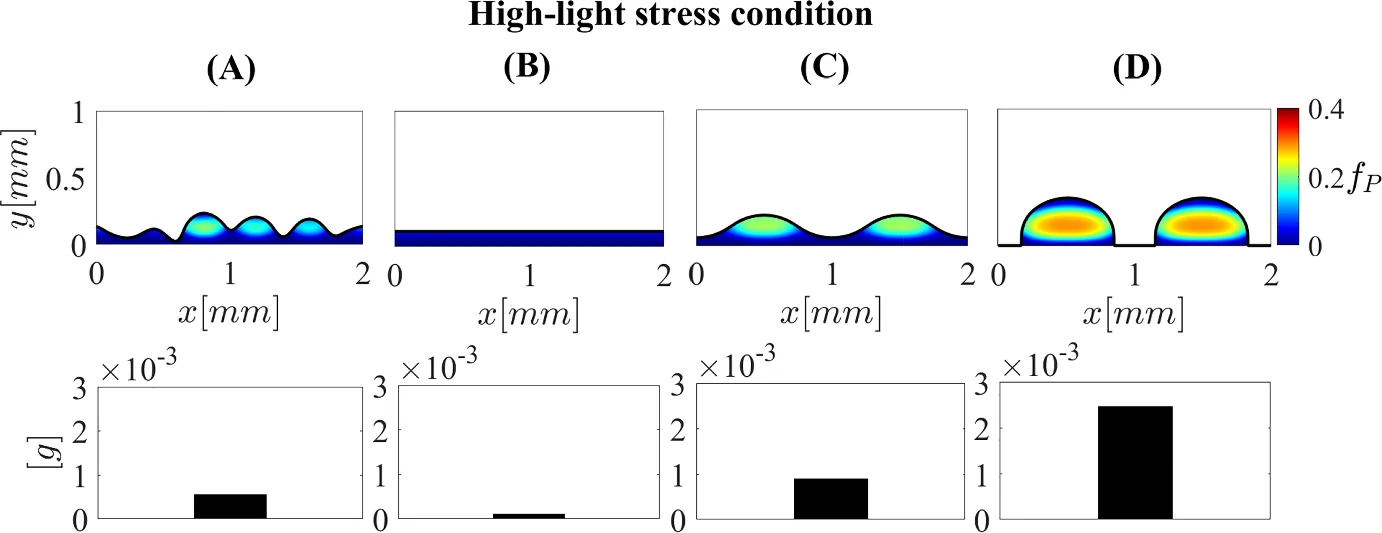
Distribution (top panels) and total mass (bottom panels) of sessile phototrophs $f_P$ within the biofilm under high-light stress conditions ($I_0=0.041$ kmol ($\hbox {e}^-$) $\hbox {m}^{-2}$ $\hbox {d}^{-1}$), at 8 days, for different initial biofilm geometries. $D_{\Psi } =$ $10^{-7}$ $\hbox {m}^2$ $\hbox {d}^{-1}$, $\chi _1 =$ $10^{-5}$ g(COD) $\hbox {m}^{-1}$ $\hbox {d}^{-1}$ (Color figure online)

**Fig. 23:**
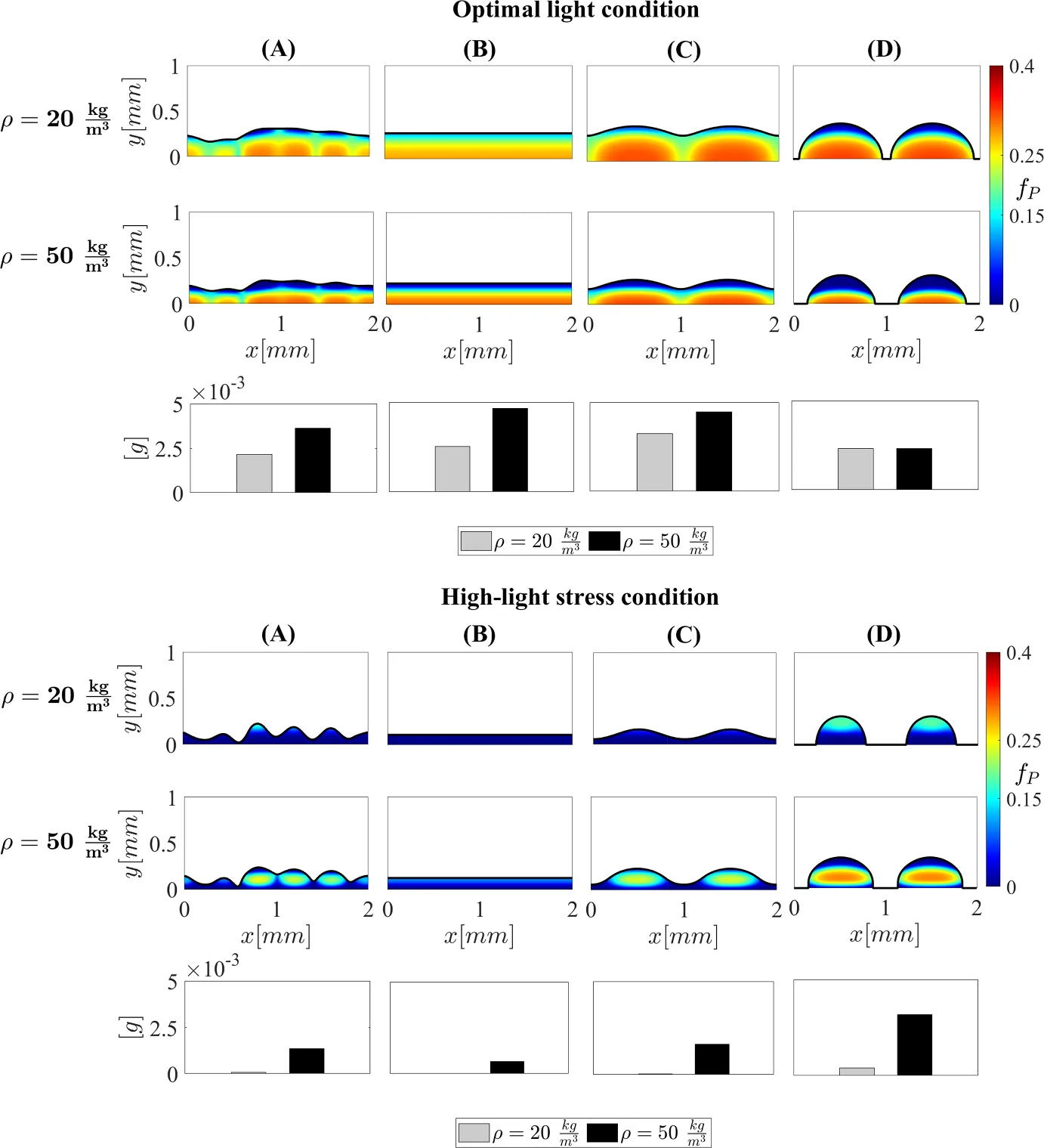
Distribution and total mass of sessile phototrophs $f_P$ within the biofilm under optimal light ($I_0=I_{\text {opt}}=0.01728$ kmol ($\hbox {e}^-$) $\hbox {m}^{-2}$ $\hbox {d}^{-1}$) and high-light stress conditions ($I_0=0.041$ kmol ($\hbox {e}^-$) $\hbox {m}^{-2}$ $\hbox {d}^{-1}$), at 8 days, for different biofilm density values and initial biofilm geometries. The light intensity is set at $I_0=I_{\text {opt}}=0.01728$ kmol ($\hbox {e}^-$) $\hbox {m}^{-2}$ $\hbox {d}^{-1}$. $D_{\Psi } =$ $10^{-7}$ $\hbox {m}^2$ $\hbox {d}^{-1}$, $\chi _1 =$ $10^{-5}$ g(COD) $\hbox {m}^{-1}$ $\hbox {d}^{-1}$ (Color figure online)

The three scenarios examined in the previous studies—concerning the effects of random and tactic motility, high-light stress conditions, and biofilm density, respectively—are analyzed here for the different initial biofilm geometries. Specifically, Figs.  21, 22 and 23 show the biofilm growth and the spatial arrangement of sessile phototrophs (top panels), together with the total sessile biomass (bottom panels).

Figure 21 confirms the key role of tactic motility in promoting the proliferation of sessile phototrophic biomass, regardless of the initial biofilm geometry. Indeed, all the considered configurations exhibit a substantial increase in biomass when tactic motility is enabled compared with the random motility case. Nevertheless, significant quantitative differences emerge among the different geometries, which are primarily governed by light availability within the biofilm. In biofilm geometries (B) and (C), light attenuation is less pronounced owing to their more favorable morphology, resulting in a more uniform and overall higher light intensity throughout the biofilm. Consequently, these configurations achieve the highest phototrophic biomass. Conversely, biofilm (A) exhibits a more irregular structure, with regions located farther from the light source where attenuation effects become more severe, ultimately leading to a lower overall biomass production. This trend becomes even more pronounced for biofilm (D), whose geometry further intensifies light attenuation, limiting light availability over larger portions of the biofilm and resulting in the lowest biomass accumulation among the investigated cases.

Notably, narrow vertical channels characterized by a low sessile phototroph concentration emerge in geometries (A) and (C). This behavior arises because the thinner regions of the initial biofilm receive a reduced supply of planktonic cells, resulting in limited phototrophic biomass growth and, consequently, the persistence of these low-concentration channels. In contrast, such channels do not form when the initial biofilm is more uniform along the x-direction, as in geometry (B). In this configuration, the biofilm thickness is sufficiently large throughout the domain to sustain the accumulation of planktonic cells, thereby promoting a more homogeneous development of the phototrophic biomass.

The spatial arrangement observed in Fig.  21 for all the investigated geometries is obtained under the optimal light condition, i.e., $I_0=I_{\text {opt}}$, and can be reasonably extended to light regimes with $I_0<I_{\text {opt}}$, as photoinhibition does not occur and, consequently, negative phototaxis is not triggered. Under these conditions, phototrophic cells preferentially migrate toward regions of higher light availability, leading to the spatial distributions discussed above. Conversely, when the incident light intensity exceeds the optimal value, the biofilm organization changes substantially. This behavior is illustrated in Fig.  22, which reports the same model outputs under tactic motility with $I_0 = 0.041$ kmol ($\hbox {e}^-$) $\hbox {m}^{-2}$ $\hbox {d}^{-1}$, which exceeds $I_{\text {opt}}$. The distribution of sessile phototrophic biomass shifts toward the upper biofilm layers, farther from the light source, where light attenuation reduces the local light intensity toward values closer to the optimum for growth. As a result, the impact of the initial biofilm geometry is reversed. In particular, geometry (D), which was the least favorable configuration under optimal light conditions, becomes the most advantageous, whereas geometry (C), previously associated with the highest biomass production, becomes the least favorable. This behavior reflects the different light attenuation patterns generated by each biofilm morphology: geometries that promote stronger attenuation provide a larger portion of the biofilm with light conditions closer to the optimal value, thereby enhancing phototrophic growth under high incident light intensities.

Finally, Fig.  23 reports the results obtained for two different values of ρ under the two light conditions previously discussed, considering all the investigated biofilm geometries. Also in this case, the effects of ρ in combination with light conditions are qualitatively consistent across all the considered biofilm configurations with those described in the previous section. The observed quantitative differences are again mainly governed by the interaction between biofilm geometry and light attenuation.

### Overview of Parameter Effects on Planktonic Phototrophs

**Table 2:** Sensitivity indices of the model parameters with respect to the mass of planktonic phototrophs for each pairwise parameter study

${\chi _1-D_{\Psi }}$		${\chi _1-k_{col}}$		${\chi _1-\chi _2}$		${\mu _{max,H}-\mu _{max,P}}$		${\chi _1-I_0}$		${\rho -I_0}$	
$\chi _1$	$D_{\Psi }$	$\chi _1$	$k_{col}$	$\chi _1$	$\chi _2$	$\mu _{max,H}$	$\mu _{max,P}$	$\chi _1$	$I_0$	ρ	$I_0$
+	–	++	+	+	–	0	++	++	++/– –	++	++/– –

The symbols + (–) indicate a positive (negative) influence of the corresponding parameter on the mass of planktonic phototrophs, while ++ (– –) denote a stronger positive (negative) influence. The symbol 0 indicates a negligible or no appreciable effect within the investigated parameter space. $\chi _1$: phototaxis constant rate; $D_{\Psi }$: diffusion coefficient of $\Psi _P$; $k_{col}$: maximum colonization rate of $\Psi _P$; $\chi _2$: phototaxis saturation constant; $\mu _{max,H}$: maximum growth rate of sessile heterotrophs; $\mu _{max,P}$: maximum growth rate of sessile phototrophs; $I_0$: incident light intensity; ρ: biofilm density

To provide a concise overview of the effects of the parameters investigated in the pairwise parametric studies (reported in Figs. 9, 10, 11, 12, 16, and 19), we assessed their influence on the mass of planktonic phototrophs $m_{\Psi }$, which was selected as the reference output because it provides an overall measure of the initial establishment of phototrophic biomass. For each pair of parameters investigated $(p_1,p_2)$, two average sensitivity indices were computed:

18 $$\begin{aligned} \overline{SI}_{p_1}= \frac{1}{n_1 n_2} \sum _{i=1}^{n_1} \sum _{j=1}^{n_2} \left| SI_{p_1}(p_{1,i},p_{2,j}) \right| , \qquad \overline{SI}_{p_2}= \frac{1}{n_1 n_2} \sum _{i=1}^{n_1} \sum _{j=1}^{n_2} \left| SI_{p_2}(p_{1,i},p_{2,j}) \right| , \end{aligned}$$

where $SI_{p_1}$ and $SI_{p_2}$ represent the sensitivity indices:

19 $$\begin{aligned} SI_{p_1}= \frac{\partial m_{\Psi }(p_{1,i},p_{2,j})}{\partial p_1} \frac{p_1}{m_{\Psi }(p_{1,i},p_{2,j})}, \quad SI_{p_2}= \frac{\partial m_{\Psi }(p_{1,i},p_{2,j})}{\partial p_2} \frac{p_2}{m_{\Psi }(p_{1,i},p_{2,j})}. \end{aligned}$$

Here, $n_1$ and $n_2$ denote the number of investigated values of the parameters $p_1$ and $p_2$, respectively, within the corresponding pairwise parameter space.

According to predefined thresholds, the results were classified into five categories, denoted by the symbols ++, +, 0, –, and – –, representing strong positive, positive, negligible, negative, and strong negative effects, respectively. The intensity of the effect was quantified based on the magnitude of the average sensitivity indices $\overline{SI}_{p_1}$ and $\overline{SI}_{p_2}$: values < 1 were classified as moderate effects (+ or –), while values > 1 were classified as stronger effects (++ or – –). Sensitivity values close to zero ($\overline{SI}_{p_k} \le 0.1$), corresponding to negligible variations of the mass of planktonic phototrophs within the investigated parameter space, were classified as 0. The sign of the effect was assessed by evaluating the minimum and maximum values of the sensitivity index $SI_{p_k}$ over the investigated parameter space, where k=1,2. When minimum and maximum values exhibited opposite sign, both symbols were assigned (e.g., +/– or ++/– –) to indicate that the parameter effect was not univocal and depended on the specific combination of parameter values. The resulting classification provides an at-a-glance overview of both the direction and the relative importance of the parameter effects, complementing the detailed information conveyed by the heatmaps showed in the previous sections.

The results, reported in Table 2, highlight the different roles played by the investigated parameters in controlling the mass of planktonic phototrophs. The phototaxis constant rate $\chi _1$ consistently exhibits a positive influence and emerges as one of the most relevant parameters. However, its sensitivity magnitude varies depending on the paired parameter, reflecting the coupled and nonlinear interactions among the processes governing planktonic dynamics. In particular, $\chi _1$ shows a stronger effect when coupled with $k_{col}$ and $I_0$. The diffusion coefficient of $\Psi _P$, $D_{\Psi }$, and the phototaxis saturation constant $\chi _2$ both exhibit a moderate negative influence on planktonic phototrophs, respectively associated with increased cell dispersion and spatial saturation effects. Conversely, the maximum colonization rate of $\Psi _P$, $k_{col}$, shows a moderate positive influence, reflecting its role in promoting biofilm expansion and, consequently, increasing planktonic cells accumulation. Among the growth-related parameters, $\mu _{max,P}$ has a strong positive influence, whereas $\mu _{max,H}$ produces negligible variations within the explored parameter range, reflecting the distinct roles played by phototrophic and heterotrophic microorganisms in the system. The incident light intensity $I_0$ exhibits both strong positive and strong negative effects, as the investigated range spans suboptimal and photoinhibitory light conditions, which induce positive and negative phototactic responses, respectively. Finally, the biofilm density ρ consistently shows a strong positive influence on planktonic phototrophs, as increased light attenuation sharpens light gradients and enhances phototactic accumulation.

It should be noted that, while the present pairwise studies allow the effects of individual parameter combinations to be assessed, a global sensitivity analysis would be required to fully quantify the relative importance and interactions of all model parameters across the entire parameter space.

## Discussion

The model introduced in this work allows for an accurate representation of the spatial distribution and growth patterns of microbial populations in response to light conditions. Our numerical results unravel the crucial role of phototaxis in shaping phototrophic biofilm’s dynamics. Planktonic cells migrate toward illuminated areas within the biofilm, accumulating where light intensity is optimal, which in turn accelerates the growth of sessile phototrophs. This light-driven movement enhances biofilm development and expansion. Phototaxis also impacts metabolic activity, as increased oxygen production by phototrophs favors heterotrophic carbon consumption, highlighting the complex interplay between microbial species. The interplay between random diffusion and phototactic movement regulates phototrophic biomass distribution. Low diffusion promotes phototactic accumulation, while high diffusion causes widespread cell dispersion. These findings highlight the large influence of motility parameters on microbial spatial organization. The optimal light condition was chosen as a baseline, representing the light intensity at which cell growth is maximized. Under these conditions, the domain’s geometry results in unidirectional movement of planktonic cells, induced by the vertical monotony of the phototactic function. For conditions above the optimal light intensity, the model simulates environments where light can become detrimental to cellular function, potentially leading to photoinhibition or damage to the cells. In these cases, photoinhibition reduces phototrophic growth and shifts phototaxis from positive to negative. This causes planktonic cells to move away from the light source, and leads to an overall slower biofilm development. In addition, the model results suggest that biofilm density might control light penetration, shaping phototaxis. Higher biofilm densities are associated with increased light attenuation, leading to reduced light availability for cells within deeper biofilm layers. However, numerical results suggest that this is not necessarily disadvantageous for phototrophic bacteria.

In summary, our analysis highlights the complex interplay between biofilm density and geometry, light conditions, and phototactic behavior, contributing to a deeper understanding of the factors that influence microbial population dynamics in phototrophic biofilms. The model simulations indicate that phototaxis has a profound impact on biofilm composition and growth. From an engineering perspective, this is particularly relevant to the start-up phase of biofilm-based photobioreactors, where promoting rapid biofilm establishment is essential for efficient reactor operation. In this context, the model provides experimentally testable predictions on the effect of phototactic migration during the early stages of biofilm formation, which could be validated under controlled illumination conditions by monitoring the spatial distribution of biomass over time. Furthermore, since quantitative data for the motility-related parameters are currently unavailable in the literature, the performed parametric analysis identifies the ranges of parameter values for which phototaxis significantly affects biofilm development. This provides a framework for future experimental calibration of the model and for estimating the motility properties of different phototrophic strains. Once calibrated, the model could support the selection of inocula with favorable phototactic characteristics and the identification of operating conditions that promote efficient biofilm establishment during reactor start-up. While these findings highlight the potential of the proposed modeling framework, they should be interpreted in light of the simplifying assumptions adopted in the present study, which also indicate several directions for future model development and experimental investigation.

In this study, we assume the biofilm to be a homogeneous medium with uniform light attenuation properties. This assumption allows to capture general trends and draw qualitative conclusions about phototaxis and biofilm dynamics. However, it is important to note that different microbial species within the biofilm may play distinct roles in light attenuation. Future experimental studies that focus on the specific attenuation characteristics of different microbial species would provide more precise insights into this phenomenon. Incorporating species-specific light attenuation into the modeling framework therefore represents a natural extension of the present work, further strengthening its predictive capability and applicability to real biofilm systems.

To account for cell movement, our model allows planktonic cells to move both phototactically and randomly, whereas sessile cells are considered immobile. Although this assumption is reasonable for light-controlled systems, such as photobioreactors, some studies suggest that also sessile cells in natural environments may adjust their positions in response to the daily light fluctuations (Kingston 1999; Consalvey 2002; Consalvey et al. 2004). This movement could serve as an adaptive strategy to sustain photosynthesis and minimize the photoinhibitory effects of high irradiance, thereby enhancing overall fitness (Mouget et al. 2008). Future research on the dynamics of sessile cell movement would be valuable for refining this biofilm model and advancing our understanding of biofilm ecology in dynamic natural environments.

It is important to note that some of the qualitative conclusions drawn from the simulations may depend on the initial biofilm geometry. The choice of a randomly irregular initial biofilm geometry adopted in Sects.  3.1–3.3 was motivated by the aim of reproducing the pronounced spatial heterogeneity in thickness observed in real biofilm systems and allowed the model to capture the main features of light distribution within the biofilm. However, the results reported in Sect.  3.4 indicate that biofilm geometry can quantitatively affect biofilm dynamics. In particular, it can alter light attenuation, reshaping local light availability and, consequently, microbial responses and overall biofilm development. Thus, while the randomly irregular initial biofilm geometry provides a realistic framework for capturing the main mechanistic features governing biofilm dynamics, the choice of the initial geometry remains an important factor in shaping key system-level outcomes, phototrophic biomass production and distribution, oxygen production, and organic carbon removal, with direct implications for photobioreactor performance. Also, while the present model can track the evolution of multiple disconnected biofilm colonies through separate free-boundary components, it assumes that these colonies remain disjoint throughout the simulation. The current explicit free-boundary framework cannot accommodate the merging of initially disconnected colonies, a scenario that lies beyond the scope of the present work. Within a continuum modeling framework, there are some modeling approaches that can handle these kinds of phenomena without an explicit interface representation, such as the ones in Ghasemi et al. (2021, 2023).

Detachment and dispersal processes are not considered in the proposed model. Dispersal can be interpreted as the reverse process of microbial invasion, that is the release of planktonic cells from the biofilm into the surrounding acqueous phase, internally or externally triggered (Emerenini et al. 2015, 2016). Incorporating dispersal dynamics and modelling motility of dispersed cells as governed by taxis phenomena would be essential for studying the maturation phase of biofilm development and investigate potential effects of this phenomenon in biofilm structure.

Finally, the present model assumes purely diffusive transport in the liquid surrounding the biofilm, corresponding to hydrostatic or, more generally, low-Peclet-number conditions, under which advective transport is negligible. At higher Peclet numbers, however, external advection may significantly affect solute transport and, consequently, biofilm growth and morphology. Extending the present framework to account for liquid flow may represent an important direction for future research.

## Data Availability

The data that have been used are confidential.
